# Zinc Oxide Nanoparticles in Modern Science and Technology: Multifunctional Roles in Healthcare, Environmental Remediation, and Industry

**DOI:** 10.3390/nano15100754

**Published:** 2025-05-17

**Authors:** Veeranjaneya Reddy Lebaka, Perugu Ravi, Madhava C. Reddy, Chandrasekhar Thummala, Tapas Kumar Mandal

**Affiliations:** 1 Department of Microbiology, Yogi Vemana University, Kadapa 516005, Andhra Pradesh, India; lvereddy@yahoo.com (V.R.L.); raviperugu999@gmail.com (P.R.); 2 Department of Biotechnology and Bioinformatics, Yogi Vemana University, Kadapa 516005, Andhra Pradesh, India; cmadhavareddy@gmail.com; 3 Department of Environmental Science, Yogi Vemana University, Kadapa 516005, Andhra Pradesh, India; tcsbiotech@gmail.com; 4 School of Chemical Engineering, Yeungnam University, Gyeongsan 38541, Republic of Korea

**Keywords:** zinc oxide nanoparticles (ZnO NPs), multifunctional nanomaterials, biomedical applications, nanotechnology in agriculture, environmental remediation

## Abstract

Zinc oxide nanoparticles (ZnO NPs) have garnered significant attention across various scientific and technological domains due to their unique physicochemical properties, including high surface area, photostability, biocompatibility, and potent antimicrobial activity. These attributes make ZnO NPs highly versatile, enabling their application in biomedicine, environmental science, industry, and agriculture. They serve as effective antimicrobial agents in medical treatments and as catalysts in environmental purification processes, owing to their ability to generate reactive oxygen species (ROS) and exhibit photocatalytic activity under UV light. Moreover, ZnO NPs are being increasingly employed in advanced drug delivery systems and cancer therapies, highlighting their potential in modern medicine. Their growing popularity is further supported by their ease of synthesis, cost-effectiveness, and capacity for diverse functionalization, which expand their utility across multiple sectors. This review focuses on research from the past five years (2020–2025) on the practical uses of ZnO nanoparticles in the biomedical, environmental, industrial, and agricultural fields. It also highlights current trends, existing challenges, and future perspectives. By examining these aspects, the article provides a comprehensive understanding of the versatile roles of ZnO NPs and their emerging significance in science and technology.

## 1. Introduction

Zinc oxide nanoparticles (ZnO NPs) have garnered significant attention across various scientific and technological domains due to their distinctive physicochemical properties. Their high surface area, photostability, biocompatibility, and strong antimicrobial activity enable a broad spectrum of applications in the biomedical, environmental, industrial, and agricultural fields [1]. The increasing interest in ZnO NPs is further fueled by their ease of synthesis, cost-effectiveness, and versatile functionalization capabilities, enabling customized applications across diverse sectors [2]. ZnO NPs are particularly recognized for their potential in medical applications, where their antimicrobial properties are leveraged to combat a broad range of pathogens [3]. Their ability to generate reactive oxygen species (ROS) under UV light, along with their photocatalytic activity, further enhances their utility in environmental applications such as water purification and air pollution control [4]. Moreover, the incorporation of ZnO NPs into drug delivery systems and cancer therapies underscores their emerging role in advancing modern medicine [5]. This review aims to present a comprehensive overview of recent advancements in the application of ZnO nanoparticles, with a particular focus on studies published in the past few years. It explores their roles across various sectors—including biomedical, environmental, industrial, and agricultural—while intentionally excluding synthesis methodologies to emphasize practical implementations. The objective is to highlight the current trends, existing challenges, and future prospects of ZnO NPs in these diverse fields.

### 1.1. Scope and Objectives of the Review

➢ To provide a comprehensive overview of the recent advancements in the synthesis, characterization, and multifunctional applications of zinc oxide nanoparticles (ZnO NPs) from 2020 to 2025.
➢ To explore the diverse roles of ZnO NPs in biomedical applications, including antimicrobial activity, targeted drug delivery, cancer therapy, and vaccine development.
➢ To highlight the significant contributions of ZnO NPs in environmental remediation, such as water purification, pollutant degradation, and sustainable waste management.
➢ To examine the use of ZnO NPs in industrial sectors like electronics, optoelectronics, packaging, and textiles, focusing on their optical, electrical, and antimicrobial properties.
➢ To assess the potential of ZnO NPs in agricultural innovation, including their use as nanofertilizers, nanopesticides, and growth-promoting agents.
➢ To discuss the toxicological and safety aspects associated with ZnO NPs, emphasizing their impact on human health and ecosystems.
➢ To identify the challenges, limitations, and future perspectives that may guide the development of safer and more efficient ZnO-based nanotechnologies.
➢ To support interdisciplinary collaboration by bridging nanoscience with healthcare, agriculture, environmental engineering, and industrial technology.

### 1.2. Review Methodology

To provide a comprehensive overview of the current advancements and applications of zinc oxide nanoparticles (ZnO NPs), a systematic literature review was conducted. The information for this review was gathered through extensive searches of reputable scientific databases, including PubMed, Web of Science, and Scopus, ensuring access to high-quality, peer-reviewed articles.

A combination of specific keywords, including “ZnO nanoparticles”, “applications”, “antimicrobial”, “photocatalytic activity”, “biomedical applications”, “environmental remediation”, “drug delivery”, and “nanomaterials”, was employed to identify relevant studies published between 2020 and 2025. The inclusion criteria focused on articles that discussed the functional properties, synthesis, and practical applications of ZnO NPs across various fields such as biomedicine, environmental science, and industrial processes. Articles were selected based on their relevance to the topics of antimicrobial activity, photocatalysis, environmental purification, and nanomedicine. The selected studies were critically analyzed, with an emphasis on recent advancements, challenges, and emerging trends within each application domain. Furthermore, only research articles, reviews, and studies that reported original experimental data or comprehensive meta-analyses were considered. A rigorous screening process was applied to ensure that only high-quality and relevant publications were included in this review. This methodology ensured that the findings presented in this review reflect the most current and comprehensive information available on the versatile applications of ZnO NPs in diverse scientific and technological fields.

## 2. Applications of Zinc Oxide Nanoparticles

### 2.1. Biomedical Applications

#### 2.1.1. Antimicrobial Activities

Zinc oxide nanoparticles (ZnO NPs) have demonstrated substantial potential in combating a wide range of microbial infections, owing to their distinctive mechanisms of action. These include the generation of reactive oxygen species (ROS), disruption of microbial cell membranes, and inhibition of biofilm formation, a protective barrier that enhances microbial resistance to treatment. This section examines the antibacterial, antifungal, and antiviral applications of ZnO NPs reported between 2021 and 2024.

##### Antibacterial Activity

Zinc oxide nanoparticles (ZnO NPs) have emerged as a potent tool in combating multidrug-resistant (MDR) bacteria, which represent a major challenge in healthcare due to their involvement in hospital-acquired infections (HAIs). ZnO NPs effectively target pathogens such as *Staphylococcus aureus* and *Escherichia coli* by attaching to bacterial cell walls, penetrating them, and inducing cell death. Moreover, their ability to generate reactive oxygen species (ROS) further disrupts bacterial structures, amplifying their antibacterial efficacy [6]. To improve their antibacterial efficacy, ZnO nanoparticles have been combined with other nanoparticles, such as silver (Ag) NPs, to form hybrid materials with an enhanced antimicrobial performance. These hybrid nanostructures exhibit synergistic effects, making them particularly effective against both Gram-positive and Gram-negative bacteria through the integration of multiple antimicrobial mechanisms [7]. The morphology of zinc oxide nanoparticles (ZnO NPs) plays a pivotal role in determining their physicochemical and biological properties, especially in antimicrobial applications. Variations in size, shape, and surface area significantly influence their reactivity and interaction with microbial membranes [8]. ZnO NPs can also play a crucial role in wound healing, where they are incorporated into dressings to prevent bacterial colonization and promote the natural healing process by stimulating cell growth and reducing inflammation. These applications are especially beneficial for chronic wounds, such as diabetic ulcers [9]. Beyond healthcare, ZnO NPs can contribute to food preservation by incorporation into packaging materials, where they inhibit bacterial growth on food surfaces, thus extending shelf life and reducing spoilage [10,11].

**Figure 1:**
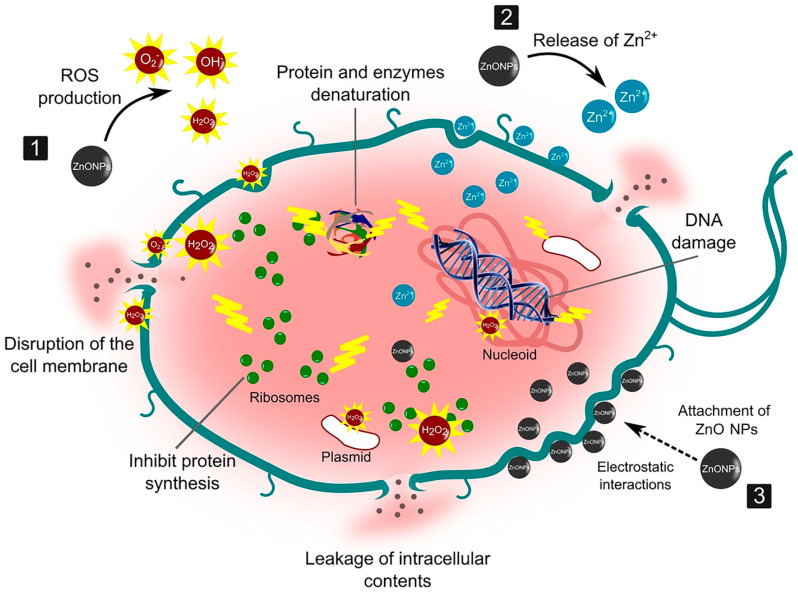
Schematic representation of the antibacterial mechanisms of zinc oxide nanoparticles (ZnO NPs). The antimicrobial effects of ZnO NPs are attributed to: (1) generation of reactive oxygen species (ROS), leading to oxidative stress, disruption of cellular membranes, and DNA fragmentation; (2) release of Zn^2+^ ions, which interfere with vital metabolic pathways and enzymatic functions; and (3) electrostatic interactions between ZnO NPs and bacterial membranes, resulting in structural membrane damage and leakage of intracellular components. Permission from ref [15].

Moreover, veterinary medicine has utilized ZnO NPs in animal feed to reduce bacterial infections in livestock, thereby minimizing the need for antibiotics and helping to mitigate the risk of antibiotic resistance [12]. In textiles, ZnO NPs are integrated into hospital uniforms and bedding, providing long-lasting antibacterial protection and helping to reduce the spread of infections in healthcare settings [13]. Topical creams containing ZnO NPs have demonstrated effectiveness in burn treatment, preventing infections and promoting tissue regeneration [14]. Figure 1 illustrates the multifaceted antibacterial action of zinc oxide nanoparticles (ZnO NPs), highlighting their potential as effective antimicrobial agents. One key mechanism involves the generation of reactive oxygen species (ROS), which trigger oxidative stress in bacterial cells, ultimately damaging cellular membranes, proteins, and genetic material. In parallel, the dissolution of ZnO NPs leads to the release of Zn^2+^ ions, which interfere with essential biological processes, such as enzyme function and protein synthesis, thereby impairing cell viability. Additionally, ZnO NPs can directly interact with bacterial membranes through electrostatic attraction, disrupting membrane integrity and causing the leakage of intracellular contents. These combined effects compromise bacterial homeostasis and lead to cell death, making ZnO NPs promising candidates for combating a wide range of pathogenic microorganisms, including drug-resistant strains.

##### Antifungal Activity

**Figure 2:**
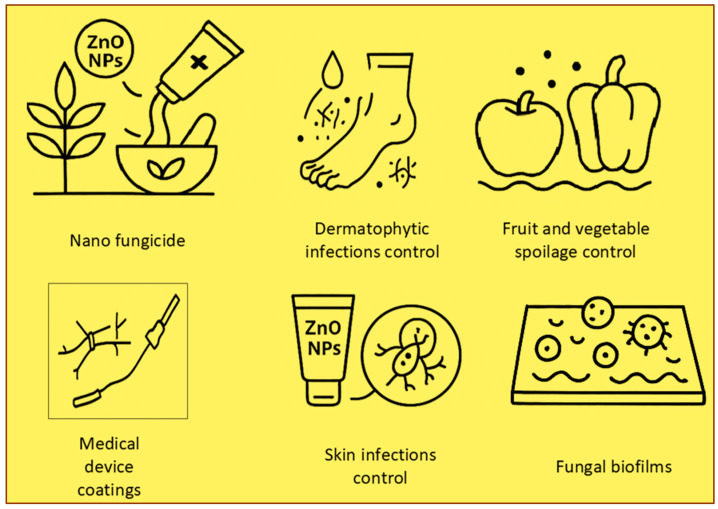
Antifungal applications of zinc oxide nanoparticles (ZnO NPs).

ZnO NPs have demonstrated potent antifungal activity against Candida albicans, a fungus responsible for infections such as oral thrush and vaginal yeast infections. By disrupting the fungal cell membrane and inhibiting growth, ZnO NPs present a promising alternative to conventional antifungal treatments, particularly for patients with drug-resistant infections [16]. In agriculture, ZnO NPs have been utilized as nanofungicides to protect crops from fungal infections. These nanoparticles are effective against a broad spectrum of plant pathogens, reducing reliance on chemical fungicides that can harm the environment. Additionally, they promote crop growth and yield by delivering essential nutrients in a controlled manner [17]. ZnO NPs are also used to treat dermatophytic infections, such as athlete’s foot, caused by fungi like Trichophyton rubrum. Applied as topical creams or powders, these nanoparticles penetrate the skin to eliminate the fungus, providing a more effective and less irritating treatment option compared to conventional antifungal creams [18]. ZnO NPs are highly effective in preventing the fungal spoilage of fruits and vegetables post-harvest. When applied as a coating on produce, these nanoparticles help to maintain freshness and extend shelf life, thereby reducing food waste and enhancing food security [19]. ZnO NPs have been integrated into polymers to develop antifungal coatings for food storage containers and packaging. These nanocomposites prevent mold and fungal growth, thereby preserving food quality and prolonging shelf life [20]. Similar to their antibacterial use, ZnO NPs have been coated on medical devices to prevent fungal infections, which can be particularly dangerous in immunocompromised patients. These coatings have been effective in reducing fungal colonization on devices like catheters and prosthetic joints [21]. ZnO NPs have also been formulated into topical gels for treating fungal skin infections, such as ringworm and jock itch. These gels provide a targeted approach to treatment, delivering the nanoparticles directly to the site of infection, where they can quickly kill fungi. Fungal keratitis, a serious eye infection caused by fungi, has been treated with ZnO NPs, showing promising results in early studies. These nanoparticles can penetrate the eye tissues and kill the fungi, offering a potential new treatment for this challenging condition [22]. Fungal biofilms, which are protective layers formed by fungi on surfaces like medical implants, are notoriously difficult to treat. ZnO NPs have shown effectiveness in disrupting these biofilms, making fungi more susceptible to treatment and reducing the risk of chronic infections [23]. In veterinary medicine, ZnO NPs have been utilized to treat fungal infections in animals, especially livestock. These nanoparticles help to reduce the prevalence of fungal diseases in herds, enhancing animal health and minimizing the economic burden of these infections on farmers [24]. Figure 2 illustrates the diverse antifungal applications of ZnO NPs across various sectors, including biomedicine, agriculture, food preservation, and veterinary medicine. It highlights the nanoparticles’ efficacy in treating fungal infections, preventing spoilage, and disrupting fungal biofilms, as well as their role in promoting crop growth and reducing environmental impacts.

##### Antiviral Activity

**Figure 3:**
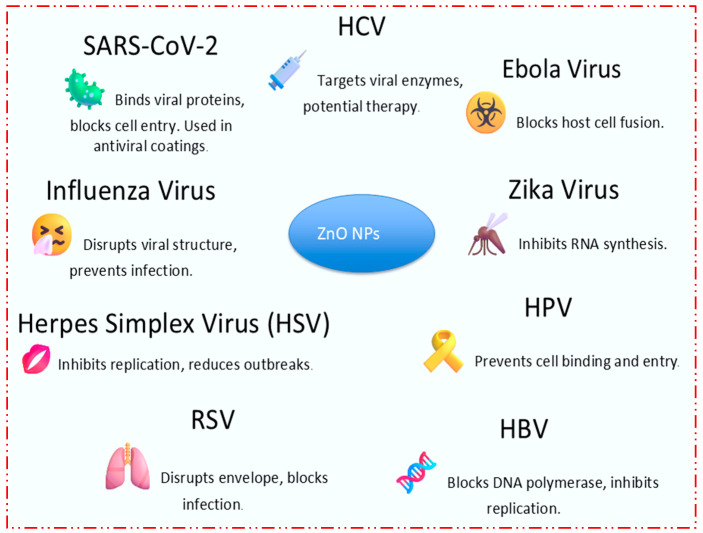
Antiviral applications of zinc oxide nanoparticles (ZnO NPs). This figure showcases the antiviral activity of ZnO NPs against a variety of viruses, including SARS-CoV-2, influenza, Herpes Simplex Virus (HSV), Respiratory Syncytial Virus (RSV), Hepatitis B Virus (HBV), Human Papillomavirus (HPV), Zika virus, and Ebola virus. It also highlights the potential use of ZnO NPs in antiviral coatings for personal protective equipment (PPE), contributing to the reduction in viral transmission.

ZnO NPs have been investigated for their potential to inhibit the replication of the SARS-CoV-2 virus, which causes COVID-19. These nanoparticles can bind to viral proteins, preventing the virus from entering and infecting human cells. This application has opened up new avenues for antiviral treatments, particularly in developing coatings for masks and surfaces that can reduce the spread of the virus [25]. ZnO NPs serve as a valuable tool in developing antiviral coatings for personal protective equipment (PPE), such as face masks and gloves. These coatings help in reducing the viral load on surfaces, making them safer for healthcare workers and the general public by minimizing the risk of viral transmission through contaminated surfaces [26]. ZnO NPs have shown significant antiviral activity against the influenza virus, which causes seasonal flu outbreaks. These nanoparticles disrupt the virus’s structure, preventing it from infecting host cells. This has implications for developing new antiviral medications and preventive measures against flu [27]. ZnO NPs have also been studied for their effectiveness against Herpes Simplex Virus (HSV), which is responsible for oral and genital herpes. These nanoparticles work by interfering with the virus’s ability to replicate, effectively reducing the severity of outbreaks. This provides a promising approach for managing Herpes Simplex Virus (HSV) infections, particularly in patients who are resistant to standard antiviral treatments [28]. ZnO NPs have also shown promise against Respiratory Syncytial Virus (RSV), a leading cause of respiratory infections in young children and elderly people. These nanoparticles inhibit the virus by disrupting its envelope, which is essential for infecting cells. This could lead to the development of new treatments for RSV, which currently has limited therapeutic options [29]. Hepatitis B Virus (HBV), which can cause chronic liver disease, is also a target for ZnO NPs. Research has shown that ZnO NPs can inhibit HBV replication by blocking the virus’s DNA polymerase enzyme, which is crucial for its reproduction. This opens up new possibilities for treating chronic HBV infections, potentially reducing the need for long-term antiviral therapy [30]. ZnO NPs have been studied for their potential to prevent infections caused by Human Papillomavirus (HPV), which is linked to cervical cancer. These nanoparticles may be used in topical formulations to prevent HPV from binding to and entering human cells, offering a preventive measure against HPV-related diseases [31]. In response to Zika virus outbreaks, ZnO NPs have been tested for their antiviral properties against this mosquito-borne virus. The nanoparticles showed the ability to inhibit Zika virus replication by disrupting its RNA synthesis. This could lead to the development of treatments that prevent the severe neurological effects associated with Zika virus infections, particularly in pregnant women [32]. ZnO NPs also find extensive use in combating Ebola virus infections, which cause severe hemorrhagic fever. These nanoparticles can inhibit the virus by blocking its ability to fuse with host cells, a critical step in the viral infection process. This research is still in its early stages, but could provide a new approach to managing Ebola outbreaks [33]. ZnO NPs have also demonstrated efficacy against Hepatitis C Virus (HCV), a major cause of liver disease worldwide. By targeting the viral enzymes necessary for HCV replication, these nanoparticles could be developed into a new class of antiviral drugs, potentially offering a cure for HCV without the severe side effects associated with current treatments [34]. Table 1 highlights the recent advancements in antimicrobial applications of zinc oxide nanoparticles (ZnO NPs) across diverse sectors, including healthcare, food safety, and environmental protection. The data demonstrate that ZnO NPs exhibit broad-spectrum antimicrobial activity against both Gram-positive and Gram-negative bacteria, such as *Staphylococcus aureus*, *Escherichia coli*, *Pseudomonas aeruginosa*, and *Salmonella typhimurium*. Their antimicrobial action is primarily attributed to the generation of reactive oxygen species (ROS), disruption of microbial membranes, and release of Zn^2+^ ions, which interfere with cellular functions. In medical applications, ZnO NPs are incorporated into wound healing gels and coatings for medical devices to prevent infections and support sterile environments. In the food industry, ZnO-based packaging materials are being explored to extend shelf life by inhibiting bacterial contamination. Additionally, textile industries are utilizing ZnO NPs for antimicrobial surface treatments to reduce microbial loads on fabrics. Their role in water purification, through UV-induced photocatalytic activity, further underscores their potential in controlling waterborne pathogens. Collectively, these multifunctional applications underscore the growing importance of ZnO NPs as effective, versatile, and eco-friendly antimicrobial agents. Figure 3 illustrates that zinc oxide nanoparticles (ZnO NPs) have garnered significant attention for their antiviral properties, offering a promising solution to combat various viral infections. The nanoparticles exhibit broad-spectrum antiviral activity by interacting with viral structures and inhibiting critical stages of the viral life cycle, such as entry into host cells and replication. For instance, ZnO NPs have been shown to prevent the SARS-CoV-2 virus from entering human cells, opening potential pathways for the development of antiviral treatments and coatings for masks and medical devices. The antiviral mechanisms of ZnO NPs are attributed to their ability to generate reactive oxygen species (ROS), disrupt viral envelopes, and block viral enzymes essential for replication. These properties make ZnO NPs a versatile tool in combating diseases caused by viruses such as influenza, HSV, RSV, and even emerging threats like the Zika virus and Ebola. Additionally, ZnO NPs’ application extends to the prevention of infections caused by HPV and HBV, offering novel approaches for prevention and treatment in clinical settings. The versatility of ZnO NPs in addressing both common and emerging viral threats highlights their potential as key components in the next generation of antiviral therapeutics and preventative measures.

#### 2.1.2. Applications of Zinc Oxide Nanoparticles in Drug Delivery

Zinc oxide nanoparticles (ZnO NPs) have emerged as versatile nanocarriers in the field of drug delivery due to their biocompatibility, ease of functionalization, and controlled release properties [64]. Their unique physicochemical characteristics make them suitable for delivering a wide range of therapeutic agents, including anticancer drugs, antibiotics, and genes, to specific target sites within the body [31]. This section explores the advancements in ZnO NPs for drug delivery from 2021 to 2024, focusing on their role in improving the efficacy and safety of treatments.

##### Targeted Drug Delivery

Targeted drug delivery aims to deliver therapeutic agents directly to the disease site, minimizing the effects on healthy tissues and enhancing treatment efficacy [65]. ZnO nanoparticles have been extensively studied for their potential in targeted drug delivery systems [66]. ZnO NPs have shown promise in targeted cancer therapy due to their ability to preferentially accumulate in tumor tissues through the enhanced permeability and retention (EPR) effect [67]. Recent studies have demonstrated that ZnO NPs can be conjugated with specific ligands, such as folic acid, to target cancer cells and deliver anticancer drugs like doxorubicin directly to tumor sites, reducing systemic toxicity [68]. The use of ZnO NPs in antibiotic delivery has gained attention for treating bacterial infections, especially those caused by antibiotic-resistant strains [25]. ZnO NPs can be functionalized with antibiotics to enhance their stability, bioavailability, and targeted delivery to infection sites. For instance, a 2023 study showed that ZnO NPs loaded with vancomycin exhibited enhanced antibacterial activity against methicillin-resistant *Staphylococcus aureus*. ZnO nanoparticles are also being explored for gene delivery applications. Their ability to protect nucleic acids from degradation and facilitate cellular uptake makes them suitable for delivering genes and small interfering RNA (siRNA) to target cells. Recent advancements include the development of ZnO-NP-based gene delivery systems for treating genetic disorders and cancers, with promising in vitro and in vivo results [69].

##### Controlled Release and Drug Loading

One of the key advantages of ZnO nanoparticles in drug delivery is their ability to provide the controlled release of therapeutic agents, ensuring a sustained and efficient treatment regimen [70]. ZnO NPs can be engineered to release drugs in response to specific stimuli, such as changes in pH. This is particularly useful for targeting tumors, which often have an acidic microenvironment. A recent study highlighted the use of pH-responsive ZnO NPs for the controlled release of paclitaxel in breast cancer treatment, resulting in an improved drug efficacy and reduced side effects [71]. The high surface area of ZnO NPs allows for a high drug loading capacity, which is essential for delivering sufficient therapeutic doses. Recent research has focused on optimizing the surface modification of ZnO NPs to enhance their drug loading efficiency, particularly for hydrophobic drugs like curcumin and paclitaxel. ZnO NPs can be incorporated into multifunctional drug delivery systems that combine therapeutic and diagnostic (theranostic) capabilities. These systems allow for simultaneous drug delivery and imaging, enabling the real-time monitoring of treatment progress. For example, ZnO NPs doped with iron oxide have been developed for combined chemotherapy and magnetic resonance imaging (MRI) [72].

##### Overcoming Drug Resistance

**Figure 4:**
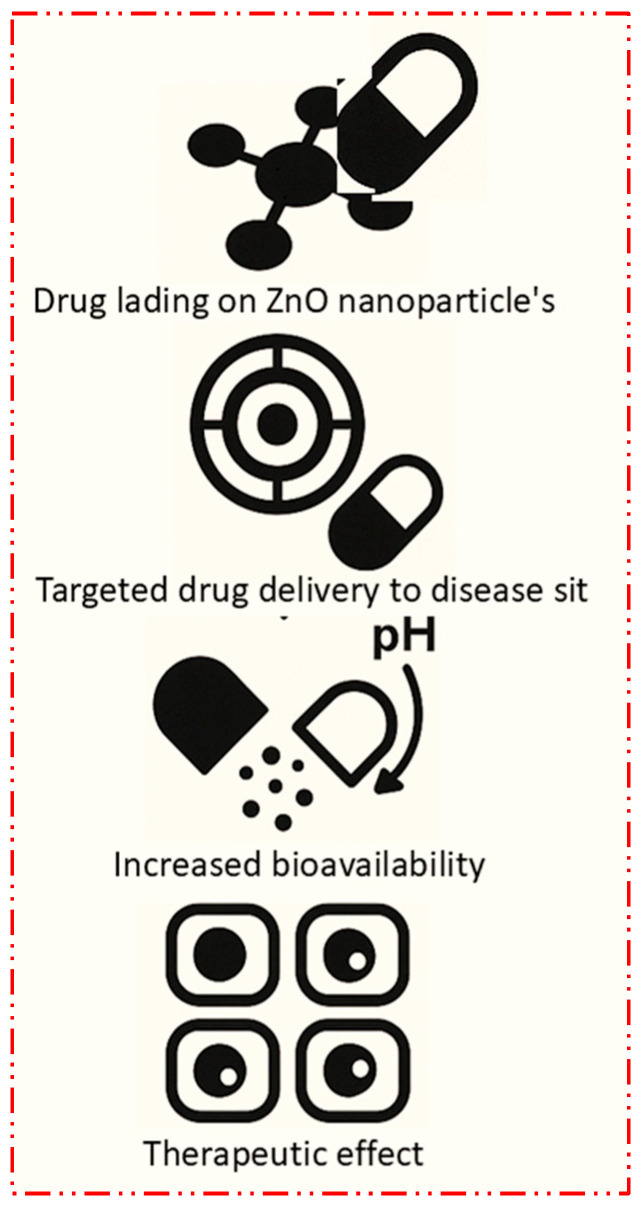
ZnO nanoparticles in overcoming drug resistance in cancer and bacterial infections.

Drug resistance remains a significant challenge in the treatment of various diseases, including cancer and bacterial infections. ZnO nanoparticles offer a potential solution by enhancing drug delivery and overcoming resistance mechanisms. ZnO NPs have been shown to reverse multidrug resistance (MDR) in cancer by inhibiting the efflux pumps that cancer cells use to expel anticancer drugs. This study demonstrated that ZnO NPs loaded with doxorubicin and verapamil (a P-glycoprotein inhibitor) successfully overcame MDR in ovarian cancer cells, leading to increased drug accumulation and enhanced cytotoxicity [73]. ZnO NPs can also enhance the efficacy of antibiotics against resistant bacterial strains by disrupting the bacterial cell membrane and facilitating drug entry. For instance, a recent study showed that ZnO NPs combined with ciprofloxacin exhibited synergistic effects against drug-resistant *Escherichia coli*, suggesting a potential strategy to combat antibiotic resistance [74]. The use of ZnO NPs in combination therapy has shown potential in addressing drug resistance. By delivering multiple therapeutic agents simultaneously, ZnO NPs can target different resistance mechanisms, improving treatment outcomes. For example, ZnO NPs co-loaded with cisplatin and curcumin have been explored for treating cisplatin-resistant lung cancer, with promising preclinical results [75]. Table 2 presents an in-depth overview of the recent multifunctional applications of zinc oxide nanoparticles (ZnO NPs) in drug delivery, underscoring their versatility in therapeutic design and precision medicine. ZnO NPs have shown great promise as nanocarriers in targeted drug delivery, particularly in cancer therapy, where their surfaces can be functionalized with targeting ligands like peptides to selectively direct drugs to tumor cells, as demonstrated in prostate cancer models. Their utility extends to antibiotic and gene delivery applications, enhancing the therapeutic potential of conventional agents and enabling nucleic acid transport into mammalian cells. ZnO NPs also exhibit pH-responsive behavior, allowing for controlled drug release specifically in acidic tumor microenvironments, which minimizes off-target effects and improves treatment efficiency. With their high surface area and modifiable surfaces, ZnO NPs can carry large amounts of hydrophobic drugs, enabling sustained release profiles. Importantly, ZnO NPs have been incorporated into multifunctional systems that integrate drug delivery with real-time imaging, improving theranostic outcomes. Furthermore, ZnO NPs address one of the most pressing challenges in modern pharmacology—drug resistance—by inhibiting the efflux pumps in cancer cells and disrupting bacterial membranes to potentiate antibiotic effects. These properties make ZnO NPs valuable in combination therapies, particularly for multidrug-resistant cancers and infections, highlighting their transformative potential in next-generation nanomedicine. Figure 4 illustrates the potential of ZnO nanoparticles (ZnO NPs) in overcoming drug resistance mechanisms. It highlights the use of ZnO NPs in combination therapies, such as doxorubicin and verapamil for ovarian cancer treatment, as well as their synergistic effects with antibiotics like ciprofloxacin in combating multidrug-resistant bacterial strains. The rising prevalence of drug resistance in both cancer and bacterial infections is a major concern in modern medicine. ZnO nanoparticles (ZnO NPs) offer a promising strategy to overcome this challenge by enhancing the efficacy of existing therapies and overcoming resistance mechanisms. In cancer treatment, ZnO NPs have been shown to reverse multidrug resistance (MDR) by inhibiting efflux pumps, which cancer cells use to expel anticancer drugs. Studies have demonstrated that ZnO NPs, when loaded with chemotherapeutic agents like doxorubicin and combined with P-glycoprotein inhibitors such as verapamil, can significantly increase drug accumulation in cancer cells and enhance cytotoxicity, overcoming resistance. Similarly, in the treatment of bacterial infections, ZnO NPs have been found to disrupt bacterial cell membranes, facilitating antibiotic entry and boosting the activity of drugs like ciprofloxacin against resistant strains of bacteria, such as *Escherichia coli*. The combination of ZnO NPs with other therapeutic agents has shown promise in simultaneously targeting multiple resistance mechanisms, improving treatment outcomes. Furthermore, ZnO NPs with functionalized surfaces have been incorporated into targeted drug delivery systems to direct drugs specifically to tumor cells, enabling enhanced precision in cancer therapy. Their pH-responsive properties also allow for controlled drug release in acidic environments like those found in tumors, reducing off-target effects and improving therapeutic efficiency. ZnO NPs are proving to be invaluable in overcoming drug resistance, both in cancer therapy and in combating antibiotic-resistant infections, marking them as essential tools in the development of next-generation nanomedicines and combination therapies.

#### 2.1.3. Applications of Zinc Oxide Nanoparticles in Cancer Treatment

**Figure 5:**
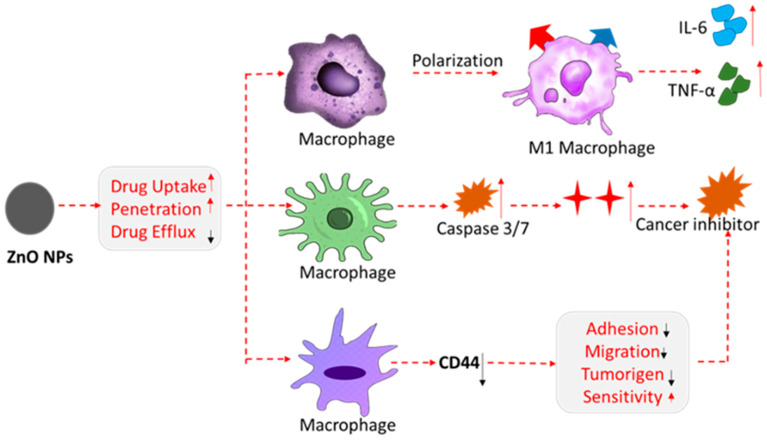
Schematic illustration of the multifunctional roles of zinc oxide nanoparticles (ZnO NPs) in cancer therapy. (Arrow color denoted: Red—increasing, black—decreasing).

Zinc oxide nanoparticles (ZnO NPs) have gained significant attention in cancer treatment due to their unique properties, such as biocompatibility, phototoxicity under UV light, and the ability to generate reactive oxygen species (ROS). These characteristics make ZnO NPs effective in targeting and destroying cancer cells while minimizing damage to healthy tissues [78]. This section explores the recent advancements in the application of ZnO NPs in cancer treatment, with a focus on studies published from 2021 to 2024. Figure 5 highlights the versatile applications of zinc oxide nanoparticles (ZnO NPs) as multifunctional agents in cancer therapy. Their anticancer activity stems from several synergistic mechanisms, including photodynamic therapy, where ZnO NPs generate reactive oxygen species (ROS) under light exposure to induce apoptosis in tumor cells. In chemotherapy, ZnO NPs enhance drug delivery by improving cellular uptake, targeting tumor tissues more precisely, and overcoming multidrug resistance by inhibiting drug efflux pathways. Furthermore, their surfaces can be functionalized for the co-delivery of multiple therapeutic agents or imaging molecules, enabling simultaneous treatment and monitoring an approach essential for personalized medicine. The ability of ZnO NPs to penetrate biological barriers, such as the tumor microenvironment or even the blood–brain barrier, further expands their therapeutic scope, making them promising nanomedicine platforms for efficient and targeted cancer management.

##### Photodynamic Therapy (PDT)

Photodynamic therapy (PDT) is a non-invasive cancer treatment that utilizes light-sensitive compounds, or photosensitizers, activated by specific wavelengths of light to produce ROS, leading to cancer cell death. ZnO nanoparticles have emerged as promising photosensitizers due to their ability to produce ROS when exposed to UV light [85]. The mechanism of action of ZnO NPs involves the generation of ROS upon UV light exposure, causing oxidative stress in cancer cells, leading to apoptosis or necrosis. Recent studies have demonstrated the efficacy of ZnO NPs in enhancing PDT’s selectivity and effectiveness in treating skin and breast cancers. The incorporation of ZnO NPs into PDT has been shown to improve the treatment’s efficacy by enhancing ROS production and reducing the required light dose. For example, a 2023 study highlighted the superior performance of ZnO NPs in treating melanoma compared to traditional photosensitizers. ZnO NPs offer the advantage of minimizing side effects by selectively targeting cancer cells while sparing healthy tissues. This selectivity is achieved by functionalizing ZnO NPs with specific ligands or antibodies that bind to cancer cell markers [86,87].

##### Chemotherapy Enhancement

ZnO nanoparticles are also being investigated for their role in enhancing the efficacy of conventional chemotherapy drugs [88]. By delivering chemotherapeutic agents directly to cancer cells, ZnO NPs help to reduce the systemic toxicity of these drugs. ZnO NPs can be conjugated with chemotherapeutic drugs like cisplatin, doxorubicin, and paclitaxel to improve their delivery and reduce off-target effects. A recent study demonstrated that ZnO NPs conjugated with doxorubicin significantly enhanced the drug’s efficacy in treating breast cancer, leading to increased cancer cell apoptosis and reduced side effects. The combination of ZnO NPs with chemotherapy drugs can result in synergistic effects, enhancing overall treatment outcomes. For example, ZnO NPs combined with cisplatin have shown enhanced cytotoxicity against lung cancer cells, suggesting a potential strategy for overcoming drug resistance. By improving drug delivery and targeting, ZnO NPs enable a reduction in the required dosage of chemotherapeutic agents, thereby minimizing side effects and improving patient outcomes [89,90].

##### Nanotheranostics

Nanotheranostics refers to the combination of therapeutic and diagnostic functionalities in a single nanoparticle system. ZnO nanoparticles are particularly suitable for nanotheranostics due to their multifunctionality, enabling simultaneous cancer treatment and imaging. ZnO NPs can be doped with imaging agents such as gold or gadolinium to create multifunctional nanoparticles that allow for the real-time monitoring of drug delivery and therapeutic response. A recent study demonstrated the effectiveness of ZnO NPs in both MRI and fluorescence imaging, along with delivering a chemotherapeutic agent to tumor sites. Nanotheranostics using ZnO NPs offers the potential for personalized cancer treatment by tailoring therapies based on individual patient profiles. The integration of diagnostic and therapeutic functions allows for the precise targeting and monitoring of cancer treatment. ZnO NPs in nanotheranostics provide a minimally invasive approach to cancer treatment, reducing the need for multiple procedures and improving patient comfort. This approach enhances the overall efficiency and effectiveness of cancer therapies [78,91]. Table 3 provides a detailed summary of the recent multifunctional applications of zinc oxide nanoparticles (ZnO NPs) in cancer treatment, emphasizing their growing potential in enhancing therapeutic efficacy and minimizing adverse effects. In photodynamic therapy (PDT), ZnO NPs act as effective photosensitizers by generating reactive oxygen species (ROS) under UV irradiation, inducing apoptosis in tumor cells. Functionalization strategies have improved the tumor-targeting capabilities and enhanced the photosensitivity of ZnO-based systems, especially when combined with natural or synthetic sensitizers. ZnO NPs also play a significant role in augmenting chemotherapy outcomes by increasing drug accumulation in resistant cancer cells, such as doxorubicin- and cisplatin-resistant lines, while concurrently reducing systemic toxicity. Furthermore, ZnO NPs enable the co-delivery of multiple drugs, facilitating synergistic effects in combination therapies. In the realm of nanotheranostics, these nanoparticles are engineered for real-time imaging and therapy through the incorporation of fluorescent markers or MRI agents, enabling dual-mode and drug-activated imaging functionalities. Notably, ZnO NPs have been tailored to target specific cancer types, such as HER2-positive breast cancer, lung adenocarcinoma, and glioblastoma, due to their ability to cross biological barriers and deliver therapeutic agents directly to tumor sites. Additionally, their excellent biodegradability and ability to reduce chemotherapy-induced side effects position ZnO NPs as a promising platform in the development of safer and more precise cancer treatments.

#### 2.1.4. Applications of Zinc Oxide Nanoparticles in Vaccine Development

Zinc oxide nanoparticles (ZnO NPs) have emerged as promising candidates in the field of vaccine development due to their ability to enhance immune responses, act as adjuvants, and serve as delivery systems for antigens. Their biocompatibility, ease of synthesis, and ability to be functionalized make them ideal for developing more effective and stable vaccines. This section discusses recent advancements in the use of ZnO NPs in vaccine development from 2021 to 2024, with a focus on their role in enhancing immune responses and providing long-term protection against various diseases [12].

##### Adjuvant Activity of ZnO Nanoparticles

ZnO NPs have been shown to enhance the immunogenicity of vaccines by serving as adjuvants, which are substances that boost the body’s immune response to an antigen. They help in the effective presentation of antigens to immune cells, thereby enhancing vaccines’ efficacy [103]. The adjuvant action of ZnO NPs can stimulate dendritic cells and macrophages, leading to the enhanced production of cytokines and an increased immune response [31]. Recent studies have demonstrated that ZnO NPs can significantly improve the immune response when used as adjuvants in vaccines against diseases such as influenza and hepatitis B [104]. A 2022 study showed that ZnO NPs used as adjuvants in a hepatitis B vaccine formulation significantly increased the production of specific antibodies, leading to enhanced protection compared to traditional adjuvants. This highlights the potential of ZnO NPs in developing more effective vaccines with longer-lasting immunity. The biocompatibility of ZnO NPs makes them suitable for use as adjuvants in human vaccines. Studies have shown that ZnO NPs are well-tolerated in the body, with minimal side effects, making them a safer alternative to traditional adjuvants [105].

##### ZnO Nanoparticles as Antigen Delivery Systems

ZnO NPs can also serve as carriers for antigens, improving the stability and delivery of antigens to the immune system. This ensures a more robust and targeted immune response, which is critical for the effectiveness of vaccines [106]. ZnO NPs can protect antigens from degradation, thereby preserving their structure and function until they reach the target site in the body. For example, a recent study demonstrated that ZnO NPs could stabilize the COVID-19 spike protein antigen, leading to a more effective immune response [107]. The surface of ZnO NPs can be functionalized with specific ligands or antibodies, allowing for the targeted delivery of antigens to specific cells or tissues [108]. This targeted approach enhances vaccines’ effectiveness by ensuring that the immune system’s response is directed precisely where it is needed. ZnO NPs can be engineered to release antigens in a controlled manner, ensuring a sustained immune response over time. This controlled release mechanism has been shown to improve the long-term efficacy of vaccines, providing extended protection against infections [109].

##### Development of New Vaccines Using ZnO Nanoparticles

**Figure 6:**
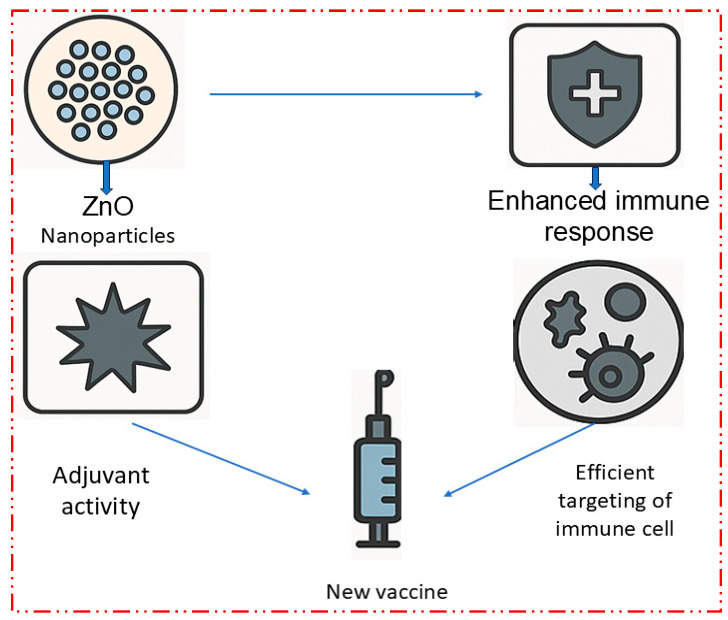
ZnO nanoparticles in vaccine development: adjuvants and antigen delivery systems.

Recent research has explored the potential of ZnO NPs in developing new vaccines for emerging and re-emerging infectious diseases. Their versatility and ability to enhance both cellular and humoral immune responses make them suitable for creating vaccines against a wide range of pathogens [58]. ZnO NPs have been investigated for use in COVID-19 vaccines due to their ability to enhance the delivery and presentation of the spike protein antigen, leading to a stronger immune response [110]. A 2023 study demonstrated that ZnO-NP-based COVID-19 vaccines induced higher levels of neutralizing antibodies compared to traditional formulations. ZnO NPs are also being explored for use in vaccines against emerging infectious diseases such as Zika virus, Ebola, and avian influenza. Their ability to enhance immune responses and provide long-term protection makes them promising candidates for these vaccines. ZnO NPs can be used to develop multivalent vaccines that target multiple antigens or pathogens simultaneously. This approach could lead to more comprehensive protection against diseases that involve multiple strains or variants, such as influenza [111,112]. Table 4 outlines the diverse and emerging roles of zinc oxide nanoparticles (ZnO NPs) in vaccine development, highlighting their multifunctional capabilities as both adjuvants and antigen delivery systems. As adjuvants, ZnO NPs have demonstrated the ability to enhance immune responses by stimulating cytokine production and promoting stronger and longer-lasting immunity, as observed in influenza vaccine studies. Their favorable biocompatibility profile also reduces the risk of adverse effects, making them suitable for preclinical and potentially clinical use. In addition to immunostimulation, ZnO NPs contribute significantly to antigen delivery systems by protecting protein-based antigens from enzymatic degradation, thereby improving stability and shelf life. Functionalization with targeting ligands enables ZnO NPs to deliver antigens directly to lymphatic tissues, enhancing immune specificity and response. Furthermore, their capacity for controlled release ensures a sustained antigen presence in the body, promoting prolonged immune activation. Recent advances have also explored the utility of ZnO NPs in next-generation vaccines, including mRNA-based COVID-19 vaccines and multivalent formulations targeting multiple pathogens simultaneously. These developments underscore the potential of ZnO NPs to revolutionize vaccine platforms by offering improved efficacy, targeted delivery, and enhanced safety profiles. Figure 6 illustrates the role of ZnO nanoparticles (ZnO NPs) in the development of new vaccines, highlighting their functions as both immune-enhancing adjuvants and efficient antigen delivery systems. It emphasizes their application in COVID-19, multivalent vaccines, and vaccines against emerging infectious diseases such as Zika and Ebola.

Zinc oxide nanoparticles (ZnO NPs) have garnered attention as promising candidates in the development of next-generation vaccines, offering multiple roles in enhancing immune responses and facilitating effective antigen delivery. One of their key applications is in the design of vaccines for emerging infectious diseases, such as COVID-19, Zika virus, Ebola, and avian influenza, due to their ability to boost both cellular and humoral immune responses. In the case of COVID-19, studies have shown that ZnO-NP-based vaccines are capable of inducing higher levels of neutralizing antibodies compared to traditional formulations, providing stronger immune protection. ZnO NPs function as adjuvants by stimulating cytokine production, which not only enhances immune responses, but also promotes longer-lasting immunity. This makes them particularly valuable in combating diseases with high mutation rates, such as influenza, where they can be incorporated into multivalent vaccines to target multiple variants or strains simultaneously.

ZnO NPs also play a crucial role in antigen delivery systems. They help to protect protein-based antigens from enzymatic degradation, thereby improving stability and shelf life, making them suitable for both preclinical and clinical applications. The functionalization of ZnO NPs with targeting ligands allows for the direct delivery of antigens to lymphatic tissues, enhancing the specificity and efficiency of the immune response. Furthermore, their ability to provide controlled release ensures that antigens are sustained in the body for prolonged periods, offering continuous immune activation. The potential for ZnO NPs to be used in mRNA-based vaccines and multivalent formulations underscores their versatility and promise in revolutionizing vaccine platforms. Their combination of enhanced efficacy, targeted delivery, and improved safety profiles highlights their transformative potential in vaccine development, making them a powerful tool in the fight against both current and future infectious diseases.

### 2.2. Applications of Zinc Oxide Nanoparticles in Environmental Remediation

Zinc oxide nanoparticles (ZnO NPs) have garnered significant attention in environmental remediation due to their photocatalytic, adsorptive, and antimicrobial properties. These characteristics make them highly effective in treating various environmental pollutants, including organic contaminants, heavy metals, and microbial pathogens. This section will explore the latest advancements and applications of ZnO NPs in environmental remediation from 2021 to 2024, focusing on their role in water purification, air purification, and soil remediation.

#### 2.2.1. Water Purification

Water pollution is a pressing global issue, and the development of efficient and sustainable methods for water treatment is crucial. ZnO nanoparticles have shown great promise in this area due to their ability to degrade organic pollutants and adsorb heavy metals [121]. ZnO NPs are effective photocatalysts under UV light, facilitating the degradation of various organic pollutants, including dyes, pharmaceuticals, and pesticides, in wastewater. Recent studies have demonstrated the efficiency of ZnO NPs in degrading complex organic compounds in industrial effluents [122]. ZnO nanoparticles possess a high surface area and active sites that allow them to adsorb heavy metals such as lead (Pb), mercury (Hg), and arsenic (As) from contaminated water. Research conducted between 2021 and 2023 has shown that ZnO NPs can achieve significant removal efficiencies, making them suitable for water purification applications [123]. ZnO NPs also exhibit strong antimicrobial activity, making them useful for disinfecting waterborne pathogens, including bacteria, viruses, and protozoa. This application is particularly valuable in rural and remote areas where access to clean water is limited [124].

#### 2.2.2. Air Purification

Air pollution, particularly in urban areas, poses severe health risks. ZnO nanoparticles have been explored as potential candidates for air purification due to their photocatalytic properties, which enable the breakdown of harmful airborne pollutants. Volatile organic compounds (VOCs) are a significant class of air pollutants that contribute to smog formation and adverse health effects. ZnO NPs can degrade VOCs through photocatalysis, converting them into less harmful substances. Recent studies have highlighted the effectiveness of ZnO-based air filters in reducing VOC levels in indoor environments [125]. ZnO nanoparticles can also be incorporated into air purification systems to enhance the removal of particulate matter (PM). The nanoparticles can agglomerate with PM, facilitating their capture and removal from the air. Similar to their application in water purification, ZnO NPs have demonstrated potential in inactivating airborne pathogens, including bacteria and viruses, thus reducing the spread of infectious diseases in indoor spaces [126].

#### 2.2.3. Soil Remediation

Soil contamination by heavy metals, pesticides, and industrial waste is a significant environmental concern. ZnO nanoparticles have been investigated for their ability to remediate contaminated soils through various mechanisms. ZnO NPs can immobilize heavy metals in contaminated soils by forming stable complexes, thereby reducing their bioavailability and toxicity. This approach has been shown to be effective in remediating soils contaminated with cadmium (Cd), lead (Pb), and zinc (Zn). The photocatalytic properties of ZnO NPs also make them suitable for degrading organic pollutants in soils, such as pesticides and polycyclic aromatic hydrocarbons (PAHs). Studies conducted between 2022 and 2024 have demonstrated the potential of ZnO NPs in breaking down these persistent pollutants. In addition to remediation, ZnO NPs can improve soil quality by promoting the growth of beneficial microorganisms and enhancing nutrient availability. This dual functionality makes ZnO NPs a promising tool for sustainable agriculture and environmental restoration [127,128]. Table 5 summarizes the recent multifunctional applications of zinc oxide nanoparticles (ZnO NPs) in environmental remediation, emphasizing their critical role in addressing pollution and promoting sustainability. ZnO NPs have shown exceptional efficiency in removing organic pollutants, heavy metals, and microbial contaminants from various environmental matrices, including water, air, and soil. Their strong photocatalytic activity under UV and visible light enables the degradation of persistent organic compounds such as dyes, pharmaceuticals, and pesticides through the generation of reactive oxygen species (ROS). Additionally, ZnO NPs exhibit strong adsorption capabilities and redox properties, facilitating the removal of toxic heavy metals like lead, cadmium, and arsenic. In microbial disinfection, they disrupt bacterial cell membranes and inhibit microbial growth, making them ideal candidates for water treatment systems. The nanoscale size, large surface area, and surface reactivity of ZnO NPs also allow for easy functionalization, enhancing their selectivity and efficiency in complex environmental systems. Collectively, the multifunctional roles of ZnO NPs in pollutant degradation, metal ion adsorption, and microbial control demonstrate their growing importance as eco-friendly and cost-effective nanomaterials for environmental cleanup technologies.

### 2.3. Industrial Applications of Zinc Oxide Nanoparticles

Zinc oxide nanoparticles (ZnO NPs) have found extensive applications in various industrial sectors due to their unique properties, including high surface area, UV-blocking ability, antimicrobial activity, and excellent thermal and chemical stability. This section explores the latest industrial applications of ZnO NPs from 2021 to 2024, with a focus on their use in electronics, textiles, cosmetics, and food packaging.

#### 2.3.1. Electronics and Optoelectronics

ZnO nanoparticles have become integral components in the electronics and optoelectronics industries, owing to their semiconducting properties and ability to efficiently generate and transport electrons. Their application in these fields has expanded significantly in recent years. ZnO NPs are widely used in the production of transparent conductive films, which are essential components of displays, touch screens, and solar cells. These films offer a cost-effective alternative to traditional materials like indium tin oxide (ITO), with improved transparency and conductivity [137]. ZnO-based gas sensors have gained prominence due to their high sensitivity and selectivity towards various gases, including nitrogen dioxide (NO_2_), carbon monoxide (CO), and volatile organic compounds (VOCs). Recent developments have enhanced the performance of ZnO-NP-based sensors, making them suitable for applications in environmental monitoring and industrial safety [138]. ZnO nanoparticles have been employed in the fabrication of high-efficiency LEDs, particularly in the ultraviolet (UV) and visible light spectra. Their unique optical properties enable the production of LEDs with a superior brightness and energy efficiency [139]. ZnO NPs are used in photodetectors for detecting UV light, which is critical in applications such as environmental monitoring, communication systems, and medical diagnostics. Recent advancements have focused on improving the sensitivity and response time of these photodetectors [140]. The piezoelectric properties of ZnO NPs have been utilized in the development of energy-harvesting devices, which convert mechanical energy into electrical energy. These devices are being increasingly used in wearable electronics and self-powered sensors [141]. ZnO nanoparticles are incorporated into flexible electronic devices, such as wearable sensors and foldable displays, due to their excellent mechanical flexibility and stability. Research has focused on enhancing the performance of these devices under various bending and stretching conditions [137]. ZnO NPs are also employed in thin-film transistors (TFTs) used in displays and other electronic devices. These transistors offer a high electron mobility and stability, making them suitable for next-generation electronics [142]. ZnO NPs have been explored for use in non-volatile memory devices, such as resistive random-access memory (RRAM). These devices offer advantages like low power consumption, a high speed, and excellent data retention [143]. ZnO NPs are used as electron transport layers in perovskite solar cells, enhancing their efficiency and stability. Recent studies have focused on optimizing nanoparticle size and morphology to improve light absorption and charge transport [144]. ZnO-nanoparticle-based coatings are applied to electronic devices to provide UV protection, improve thermal stability, and enhance durability. These coatings are particularly useful in outdoor electronics and harsh environments [145].

#### 2.3.2. Gas Sensing

Zinc oxide (ZnO) nanoparticles have garnered substantial interest in gas sensing due to their excellent semiconducting behavior, high surface area, and chemical stability. These properties enable ZnO to detect a wide range of gases, including NO_2_, CO, NH_3_, ethanol, and volatile organic compounds (VOCs), with a high sensitivity and selectivity [146,147]. The gas-sensing mechanism of ZnO nanoparticles is mainly based on changes in electrical resistance due to surface redox reactions. Oxygen adsorbed on the ZnO surface traps conduction band electrons, forming oxygen species (O⁻ and O_2_⁻). When a reducing or oxidizing gas interacts with these species, electrons are released or consumed, causing a measurable change in resistance [148]. These characteristics enable ZnO to effectively interact with various gas molecules, resulting in measurable changes in electrical properties, particularly resistance. The gas-sensing behavior of ZnO is predominantly governed by surface adsorption and redox reactions. In ambient air, oxygen molecules adsorb onto the ZnO surface and extract electrons from its conduction band, forming ionized oxygen species (such as O_2_^−^, O^−^, and O^2−^). This process creates a surface depletion layer, increasing the material’s resistance. The reaction can be summarized as follows:

- At low temperatures (<100 °C):

O_2_(gas) → O_2_(ads)

- At moderate temperatures (100–300 °C):

O_2_(ads) + e−→ O_2_(ads)

O_2_^−^(ads) + e^−^ →2O^−^(ads)

When a target gas (reducing or oxidizing) comes into contact with the surface, it reacts with these adsorbed oxygen species, altering the electron density and, hence, the electrical resistance of the material, as follows:

- Reducing gases (e.g., CO, NH_3_, H_2_, and ethanol):

These donate electrons back to the conduction band by reacting with surface oxygen species, decreasing resistance.

CO + O^−^→ CO_2_ + e^−^

- Oxidizing gases (e.g., NO_2_):

These further trap electrons, increasing the depletion layer and increasing resistance.

Several key parameters determine the effectiveness of ZnO NPs as gas sensors, as follows:

- Sensitivity: Degree of change in electrical signal per concentration of gas.
- Selectivity: Ability to distinguish a specific gas in the presence of other interfering gases.
- Response Time: Time taken to reach 90% of the total resistance change.
- Recovery Time: Time taken for the sensor to return to baseline after gas removal.
- Operating Temperature: ZnO sensors often operate at elevated temperatures (150–350 °C), though recent efforts focus on room-temperature operation.

To enhance gas-sensing performance, various strategies have been adopted, as follows:

1. Morphology Control:

Tailoring ZnO into nanorods, nanowires, nanosheets, and hollow spheres increases surface area and gas diffusion efficiency [149]

2. Doping with Metal Ions:

Incorporating dopants like Sn, Al, Co, and noble metals (e.g., Pt, Ag, and Au) improves sensitivity and selectivity by modifying charge carrier concentration and catalytic activity [150].

3. Formation of Heterojunctions:

ZnO combined with other semiconductors (e.g., SnO_2_, TiO_2_, MoS_2_, and graphene) forms heterojunctions that promote charge transfer and gas adsorption, enabling a superior performance [151].

4. UV Light Activation:

ZnO gas sensors can operate at lower temperatures or room temperature when UV light is used to activate surface reactions, which is advantageous for portable and wearable sensors.

ZnO-based gas sensors are widely applied in the following:

- Industrial safety monitoring (e.g., leak detection of combustible or toxic gases).
- Environmental pollution control (e.g., detection of NO_2_ and VOCs).
- Breath analysis and medical diagnostics (e.g., detection of acetone or ammonia in exhaled breath).
- Smart devices and Internet of Things (IoT) integrated sensing platforms.

Table 6 presents a comparative analysis of various ZnO-based gas sensors, emphasizing their sensitivity, selectivity, operating temperature, response/recovery times, and target gas detection capabilities. The data reveal that the performance of ZnO sensors is highly influenced by structural morphology, dopants, and synthesis methods. For example, nanorod- and nanosheet-based ZnO sensors often exhibit an enhanced surface area, which improves gas adsorption and sensitivity. Additionally, doping ZnO with elements like Ag, Cu, and Al significantly enhances selectivity and lowers the operating temperature, making these sensors more energy-efficient and suitable for real-world applications. Rapid response and recovery times are critical for real-time monitoring, and several ZnO-based formulations demonstrate a promising performance in this regard, particularly in detecting hazardous gases such as NO_2_, H_2_S, and ethanol. Overall, the table highlights the versatility and tunability of ZnO nanomaterials in gas-sensing technologies and underscores their potential in environmental monitoring, industrial safety, and medical diagnostics.

#### 2.3.3. Textiles

ZnO nanoparticles have been increasingly utilized in the textile industry due to their antimicrobial properties, UV-blocking capabilities, and potential for enhancing fabric durability and comfort. ZnO NPs are incorporated into fabrics to provide long-lasting antimicrobial protection against bacteria, fungi, and viruses. This application is particularly important in medical textiles, sportswear, and hygiene products [156]. ZnO nanoparticles are used in textiles to block harmful UV rays, offering protection against skin cancer and other UV-induced damage. These fabrics are ideal for outdoor clothing, tents, and sunshades [157]. ZnO NPs impart self-cleaning properties to fabrics by promoting the photocatalytic degradation of organic stains and pollutants. This application is gaining popularity in the production of curtains, upholstery, and automotive interiors [156]. ZnO nanoparticles are also incorporated into moisture-wicking fabrics to enhance their breathability and comfort. These textiles are widely used in active wear and outdoor gear [158]. ZnO NPs are also used to create flame-retardant textiles, offering workwear, home furnishings, and public transportation interiors with enhanced safety [159]. The antimicrobial properties of ZnO NPs also contribute to the development of anti-odor textiles, which prevent the growth of odor-causing bacteria. These fabrics are popular for socks, underwear, and footwear linings [160]. ZnO NPs improve the dyeability of fabrics, allowing for more vibrant and durable colors. This application is particularly useful in the fashion industry, where color fastness is essential [161]. ZnO nanoparticles are used in textiles to enhance thermal regulation, keeping the wearer cool in hot conditions and warm in cold environments. This technology is being increasingly applied in sportswear and military uniforms [162]. ZnO NPs reduce static electricity in fabrics, making them more comfortable to wear and preventing dust and lint accumulation. This feature is valuable in electronic work environments and cleanrooms. ZnO nanoparticles enhance the durability of textiles by improving their resistance to wear and tear, extending the lifespan of garments and reducing waste [163].

#### 2.3.4. Cosmetics

The cosmetic industry has embraced ZnO nanoparticles for their multifunctional benefits, including UV protection, skin-soothing properties, and antimicrobial effects. These applications have led to the development of advanced skincare and personal care products. ZnO nanoparticles are widely used in sunscreens for their broad-spectrum UV protection. Unlike chemical filters, ZnO NPs are photostable and provide protection against both UVA and UVB rays, making them suitable for daily use [164]. ZnO NPs are incorporated into anti-aging creams due to their ability to reflect UV light and prevent photoaging. These nanoparticles also promote collagen synthesis, reducing the appearance of fine lines and wrinkles [165]. The antimicrobial properties of ZnO NPs make them effective in treating acne by reducing the growth of acne-causing bacteria and soothing inflamed skin. Recent studies have highlighted the efficacy of ZnO-based formulations in managing acne vulgaris [166]. ZnO nanoparticles are also used in moisturizers to enhance skin hydration and barrier function. They help to lock in moisture and protect the skin from environmental stressors, making them ideal for dry and sensitive skin types [167]. ZnO NPs are incorporated into foundations, powders, and BB creams for their mattifying and skin-tone-evening effects. Their fine particle size ensures a smooth application and long-lasting coverage [168]. The antimicrobial properties of ZnO NPs are leveraged in deodorants to neutralize odor-causing bacteria, providing long-lasting freshness without the use of harsh chemicals [169]. ZnO nanoparticles are added to toothpaste formulations for their antibacterial properties, which help in preventing cavities, gum disease, and bad breath. They also contribute to whitening and enamel strengthening [170]. ZnO NPs are used in shampoos and conditioners for their ability to soothe the scalp, reduce dandruff, and protect hair from UV damage. Their incorporation into hair care products enhances shine and manageability [171]. ZnO nanoparticles are included in antiperspirant formulations to reduce sweat and prevent the growth of bacteria responsible for body odor. Their gentle action makes them suitable for sensitive skin [172]. ZnO NPs are also used in wound healing creams for their antibacterial and anti-inflammatory properties, which accelerate the healing process and reduce the risk of infection [173].

**Figure 7:**
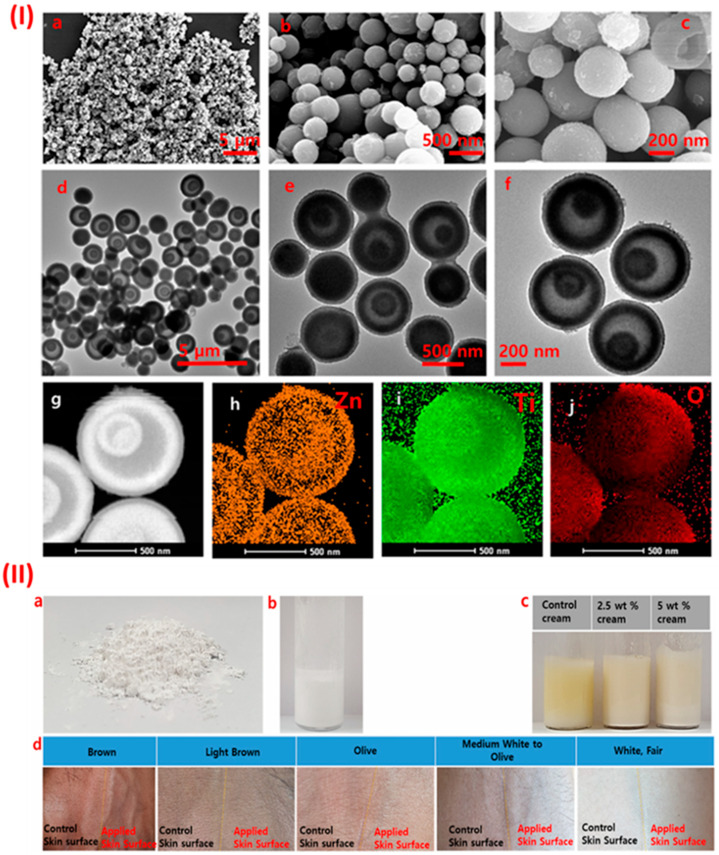
(**I**) Structural and morphological analysis of the ZnO-based composite microspheres: (**a**–**c**) Scanning Electron Microscopy (SEM) images at different magnifications; (**d**–**f**) Transmission Electron Microscopy (TEM) images at varying scales; and (**g**–**j**) Scanning Transmission Electron Microscopy (STEM) image along with corresponding elemental mapping. (**II**) (**a**) Actual photograph of TiO_2_@ZnO-PHMM powder; (**b**) visual appearance of creams formulated with 0%, 2.5%, and 5% TiO_2_@ZnO-PHMM powder dispersed in 5 mL deionized water; (**c**) close-up view of the TiO_2_@ZnO-PHMM powder; and (**d**) observation of the cream containing 5 wt% TiO_2_@ZnO-PHMM on different skin tones based on Fitzpatrick skin types. Copyright permissions from reference [174].

Figure 7 illustrates the morphological characteristics and practical application potential of ZnO and TiO_2_@ZnO-PHMM composite microspheres as multifunctional sunscreen agents. SEM and TEM images confirm the formation of uniform, well-dispersed spherical particles with nanoscale features, which are essential for effective UV scattering and absorption. STEM-EDS mapping further verifies the homogeneous distribution of Zn and Ti elements, indicating the successful integration of TiO_2_ into the ZnO matrix, which may enhance photocatalytic stability and broaden UV protection. The visual assessment of creams containing varying concentrations of TiO_2_@ZnO-PHMM demonstrates excellent dispersibility and color uniformity, with no visible aggregation. Notably, when applied to different skin tones, the 5 wt% composite cream shows minimal whitening effect, highlighting its superior aesthetic compatibility. These findings support the potential of ZnO-based metal oxide composites as advanced sunscreen materials with improved photostability, skin adherence, and broad-spectrum UV-blocking efficiency.

#### 2.3.5. Food Packaging

**Figure 8:**
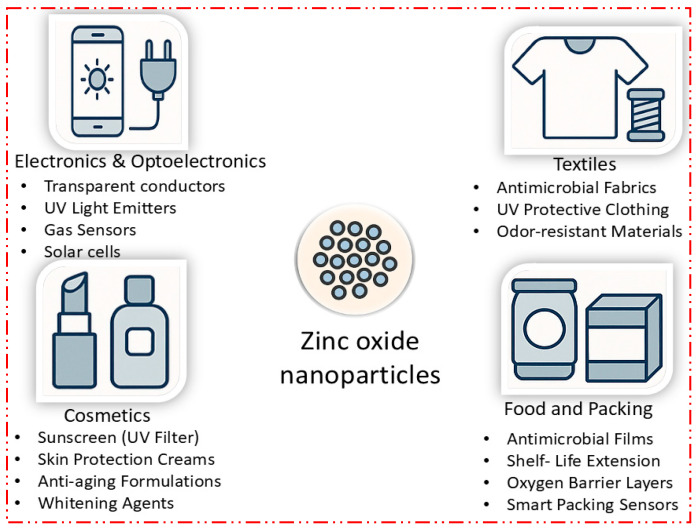
Applications of ZnO nanoparticles in active food packaging.

ZnO nanoparticles have emerged as key components in the development of active food packaging materials, which helps in extending the shelf life of food products and ensuring their safety. The antimicrobial activity of ZnO NPs is particularly effective against bacteria such as *E. coli*, *Salmonella*, and *Listeria*, making them ideal for packaging fresh produce, meats, and dairy products [175]. ZnO nanoparticles are used in food packaging to block harmful UV rays, which can cause the photodegradation of food products, leading to a loss of nutritional value and spoilage. This application is crucial for preserving the quality of light-sensitive foods, such as oils, dairy, and beverages [176]. ZnO nanoparticles can act as oxygen scavengers in packaging materials, reducing the oxygen levels inside packages and, thus, slowing down the oxidation processes that lead to rancidity and the spoilage of food products [177]. ZnO NPs are also incorporated into packaging materials to remove ethylene gas, a natural plant hormone that accelerates the ripening and spoilage of fruits and vegetables. This technology is particularly beneficial for extending the shelf life of fresh produce [178]. ZnO nanoparticles are used in the development of biodegradable packaging materials that offer active functions, such as antimicrobial activity and UV protection, while being environmentally friendly. This approach aligns with the growing demand for sustainable packaging solutions [179]. ZnO NPs are embedded in polymer matrices to create nanocomposite films with enhanced mechanical strength, barrier properties, and antimicrobial activity. These films are used in various food packaging applications, offering improved protection and an extended shelf life [180]. ZnO nanoparticles are also used in smart packaging systems that monitor and indicate the freshness of food products. These systems can change color or emit signals in response to changes in the food’s environment, such as temperature, pH, or gas composition, providing real-time information to consumers and reducing food waste [181]. ZnO nanoparticles are incorporated into packaging materials to absorb and neutralize unwanted odors released by certain foods during storage. This application is particularly useful for packaging fish, cheese, and other odor-emitting foods [180]. ZnO NPs are also added to packaging materials to enhance their barrier properties against moisture, gases, and oils. This improves the packaging’s ability to protect food from external factors, maintaining its quality and extending its shelf life [182]. ZnO nanoparticles are used in the formulation of edible coatings for fresh produce, which help in maintaining freshness, reducing microbial growth, and extending shelf life without the need for traditional packaging materials. These coatings are particularly beneficial for fruits and vegetables that are prone to spoilage [183]. Table 7 presents a comprehensive overview of the recent multifunctional applications of zinc oxide nanoparticles (ZnO NPs) in the fields of electronics, optoelectronics, and various industrial sectors. ZnO NPs have emerged as vital components in the fabrication of electronic devices due to their wide bandgap, high exciton binding energy, and excellent electron mobility. In optoelectronic applications, they are extensively used in light-emitting diodes (LEDs), photodetectors, and solar cells, where their ability to enhance light absorption and charge transport significantly improves device efficiency. Moreover, ZnO NPs are employed in sensors, transparent conductive films, and piezoelectric nanogenerators, owing to their remarkable electrical and piezoelectric properties. Industrially, ZnO NPs serve as additives in rubber and ceramic manufacturing, UV-blocking agents in coatings and paints, and catalysts in chemical processing. These diverse applications highlight the versatility of ZnO NPs in advancing modern technologies, driven by their tunable physicochemical properties and ease of integration into functional materials and devices. Figure 8 highlights the diverse applications of ZnO nanoparticles in food packaging materials, showcasing their roles in antimicrobial activity, UV protection, oxygen scavenging, ethylene removal, and smart packaging systems for extended food shelf life and improved safety. Zinc oxide nanoparticles (ZnO NPs) are being increasingly integrated into food packaging materials due to their multifunctional properties, which help to enhance food preservation, safety, and sustainability. Their antimicrobial activity is particularly significant, as ZnO NPs can inhibit the growth of harmful microorganisms such as *Escherichia coli*, *Salmonella*, and *Listeria*. This makes them ideal for packaging perishable items like fresh produce, meats, and dairy products, where microbial contamination can lead to spoilage. Beyond antimicrobial properties, ZnO NPs also offer UV-blocking capabilities, protecting food from photodegradation caused by harmful ultraviolet radiation. This is particularly important for light-sensitive products like oils, dairy, and beverages, where exposure to UV light can lead to a loss of nutritional value and quality. ZnO nanoparticles also play a vital role in extending the shelf life of food by acting as oxygen scavengers. By reducing the oxygen levels within packaging, ZnO NPs help to slow down oxidation processes that lead to rancidity in fats and oils, improving the shelf life of these products. Moreover, ZnO NPs are effective in removing ethylene gas, a natural plant hormone that accelerates the ripening and spoilage of fruits and vegetables. This technology significantly prolongs the freshness of produce, benefiting both suppliers and consumers. In response to growing environmental concerns, ZnO NPs are being incorporated into biodegradable packaging materials, offering a sustainable solution that provides antimicrobial activity and UV protection while minimizing environmental impacts. ZnO NPs are also used in the creation of nanocomposite films, which enhance the mechanical strength, barrier properties, and antimicrobial effectiveness of packaging materials. These films are a promising development for a wide range of food packaging applications. Additionally, the integration of ZnO NPs into smart packaging systems provides the real-time monitoring of food freshness. These systems can change color or emit signals in response to environmental changes, such as shifts in temperature, pH, or gas composition, offering consumers and suppliers a way to instantly gauge the quality of food products. Furthermore, ZnO NPs are effective in neutralizing unwanted odors released by certain foods during storage, such as fish and cheese, improving the overall sensory experience for consumers. With their ability to enhance food safety, shelf life, and sustainability, ZnO nanoparticles are revolutionizing the food packaging industry.

### 2.4. Agricultural Applications of Zinc Oxide Nanoparticles

**Figure 9:**
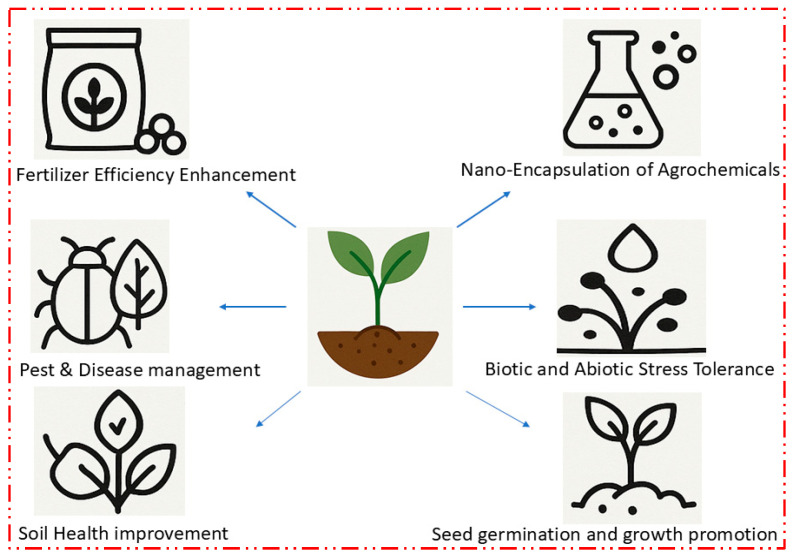
Agricultural applications of ZnO nanoparticles: enhancing Crop Growth, pest management, and soil health.

Zinc oxide nanoparticles (ZnO NPs) have gained significant attention in agriculture due to their unique properties, such as antimicrobial activity, UV protection, and the ability to enhance plant growth and nutrient uptake. These applications have the potential to revolutionize agricultural practices, improve crop yields, and reduce reliance on chemical pesticides and fertilizers. ZnO NPs have been widely studied for their antimicrobial properties, which are particularly effective against a broad spectrum of plant pathogens. These nanoparticles can be used as a protective coating for seeds, reducing the incidence of seed-borne diseases. For instance, ZnO NPs have been shown to inhibit the growth of *Pseudomonas syringae*, a common plant pathogen that affects a variety of crops [220]. This antimicrobial action is crucial for preventing crop losses and ensuring greater agricultural productivity. ZnO nanoparticles are being explored as a component of smart fertilizers, which release nutrients in a controlled manner. The nanoscale size of ZnO allows for better penetration into plant tissues, leading to more efficient zinc uptake, a critical micronutrient for plant growth [182]. Studies have shown that ZnO NPs can enhance the growth and yield of crops like wheat and maize by improving root development and increasing photosynthetic efficiency [220]. In addition to their antimicrobial properties, ZnO nanoparticles have shown potential in pest management. They can be formulated into sprays that not only protect crops from fungal and bacterial infections, but also repel insects [221]. This dual functionality makes ZnO NPs a promising alternative to conventional pesticides, which often have detrimental environmental effects [221]. ZnO NPs can play a role in improving soil health by acting as a slow-release source of zinc. This is particularly important in zinc-deficient soils, which are common in many agricultural regions. By providing a steady supply of zinc, ZnO nanoparticles help in maintaining soil fertility and supporting healthy plant growth [222]. Moreover, their small size allows for better dispersion in the soil, ensuring a more uniform nutrient availability [223]. ZnO nanoparticles can be applied as a coating on the leaves of plants to provide UV protection. This helps in reducing the damage caused by excessive UV radiation, which can lead to reduced photosynthesis and lower crop yields [224]. The application of ZnO NPs has been particularly beneficial for crops like tomatoes and strawberries, which are sensitive to UV stress [220,225]. ZnO nanoparticles have been found to enhance seed germination and early plant growth by promoting water uptake and enzyme activity in seeds [226]. This is particularly beneficial in arid regions, where water scarcity can be a major challenge. The use of ZnO NPs in seed treatments has resulted in faster germination rates and more robust seedlings, leading to improved crop establishment [227]. ZnO nanoparticles are being incorporated into materials used for soil moisture retention, helping to reduce water usage in agriculture. These materials can absorb and slowly release water, ensuring that crops receive a consistent water supply, even during dry periods [228]. This application is particularly valuable in drought-prone regions, where efficient water use is critical for sustainable agriculture [229]. ZnO nanoparticles are also being used in the nano-encapsulation of agrochemicals, such as herbicides and insecticides, to enhance their effectiveness and reduce their environmental impact. This technology allows for the targeted release of active ingredients, minimizing the amount of chemicals required and reducing runoff into surrounding ecosystems [230]. Encapsulation also protects the active ingredients from degradation, extending their effectiveness. ZnO nanoparticles have been shown to help plants tolerate biotic stresses, such as pathogen attack, and abiotic stresses, such as drought and salinity [127]. For example, the application of ZnO NPs to soybean plants was found to enhance resistance to drought by improving root structure and increasing water uptake [231]. Similarly, ZnO NPs have been used to mitigate the effects of salinity on rice crops, resulting in higher yields under saline conditions [232]. ZnO nanoparticles are being developed as components of nano-biosensors that can monitor soil health, nutrient levels, and plant stress in real time [233]. These sensors can provide farmers with precise data, allowing them to make more informed decisions about irrigation, fertilization, and pest control [234]. This technology is paving the way for precision agriculture, where inputs are optimized to maximize yield while minimizing environmental impacts [235]. Figure 9 demonstrates how zinc oxide nanoparticles (ZnO NPs) offer diverse benefits in agriculture, including antimicrobial properties, enhanced plant growth, UV protection, and improved soil health. Their use as a protective coating for seeds helps to reduce the incidence of seed-borne diseases by inhibiting plant pathogens such as Pseudomonas syringae, which is critical for preventing crop losses. ZnO NPs also contribute to more efficient nutrient uptake, especially zinc, a vital micronutrient, by acting as a component of smart fertilizers that release nutrients in a controlled manner. This leads to enhanced crop yields, as seen in crops like wheat and maize. Furthermore, ZnO NPs serve a dual function in pest management, providing protection against fungal and bacterial infections while also repelling insects, offering an eco-friendly alternative to conventional pesticides. They improve soil health by providing a slow-release source of zinc, which is essential in zinc-deficient soils, and their small size allows for better dispersion and nutrient availability. ZnO NPs can also protect crops from UV radiation, reducing stress and enhancing photosynthesis, which is particularly beneficial for UV-sensitive crops like tomatoes and strawberries. Additionally, ZnO NPs improve seed germination and early plant growth, especially in arid regions where water scarcity is a challenge, promoting faster germination and robust seedling establishment.

Table 8 summarizes the recent multifunctional applications of zinc oxide nanoparticles (ZnO NPs) in the agricultural sector, highlighting their role in enhancing crop productivity, plant protection, and sustainable farming practices. ZnO NPs serve as efficient nano-fertilizers by improving the bioavailability of zinc, an essential micronutrient involved in photosynthesis, enzyme activation, and protein synthesis, thereby promoting plant growth and yield. Their antimicrobial properties enable them to act as nano-pesticides, effectively combating bacterial and fungal pathogens without the need for excessive chemical inputs. ZnO NPs also contribute to abiotic stress tolerance in plants, helping them to withstand drought, salinity, and heavy metal toxicity through the modulation of antioxidant enzyme activity and stress-responsive genes. Moreover, their application in seed priming and foliar sprays improves germination rates and nutrient uptake efficiency. These multifunctional benefits make ZnO NPs promising tools in precision agriculture, supporting environmentally friendly and resource-efficient farming practices.

## 3. Toxicity and Safety Concerns of Zinc Oxide Nanoparticles

Zinc oxide nanoparticles (ZnO NPs) have shown immense potential across various fields, but their widespread use raises concerns about their toxicity and safety, especially regarding their impact on human health, the environment, and ecosystems. The small size and unique properties of these nanoparticles can lead to unexpected biological interactions, necessitating a thorough understanding of their toxicological profiles.

### 3.1. Human Health Risks

ZnO NPs can induce cytotoxicity in various cell lines by generating reactive oxygen species (ROS) that cause oxidative stress, leading to cell damage or death. The genotoxic potential of ZnO NPs has been demonstrated in multiple studies, where exposure has resulted in DNA damage, chromosome aberrations, and even mutations. For instance, one study showed that ZnO NPs caused significant DNA fragmentation in human lung epithelial cells, highlighting their potential genotoxic risk. The inhalation of ZnO NPs can lead to respiratory issues, including inflammation, lung damage, and fibrosis. The small size of these nanoparticles allows them to penetrate deep into the lungs, where they can induce a strong inflammatory response. Recent studies have shown that workers exposed to ZnO NPs in industrial settings have an increased risk of developing respiratory diseases, such as asthma and chronic obstructive pulmonary disease (COPD) [250,251]. ZnO NPs are widely used in sunscreens and cosmetics due to their UV-blocking properties. However, concerns have been raised about their potential to penetrate the skin and cause adverse effects. Studies have shown that while ZnO NPs generally remain on the skin surface, prolonged exposure or damaged skin can lead to penetration and subsequent toxicity, including inflammation and allergic reactions [252].

### 3.2. Environmental Impact

ZnO NPs can enter aquatic environments through wastewater discharge, leading to significant ecological consequences. These nanoparticles can be toxic to various aquatic organisms, including algae, fish, and invertebrates. For example, one study found that ZnO NPs at concentrations commonly found in wastewater could inhibit algal growth and disrupt the aquatic food chain. ZnO NPs can accumulate in soils, potentially affecting soil health and crop productivity. High concentrations of ZnO NPs have been shown to be toxic to beneficial soil microbes, which play a crucial role in nutrient cycling and plant growth [253]. One study demonstrated that exposure to ZnO NPs resulted in a significant reduction in soil microbial diversity, which could have long-term consequences for soil fertility and ecosystem stability. There is increasing concern regarding the bioaccumulation of ZnO NPs in the food chain. When these nanoparticles are absorbed by plants or ingested by smaller organisms, they can accumulate in tissues and move up the food chain, potentially impacting higher trophic levels, including humans. Research has shown that ZnO NPs can bioaccumulate in fish and other aquatic organisms, leading to toxic effects and raising concerns about food safety [254].

### 3.3. Safety Measures and Regulations

**Figure 10:**
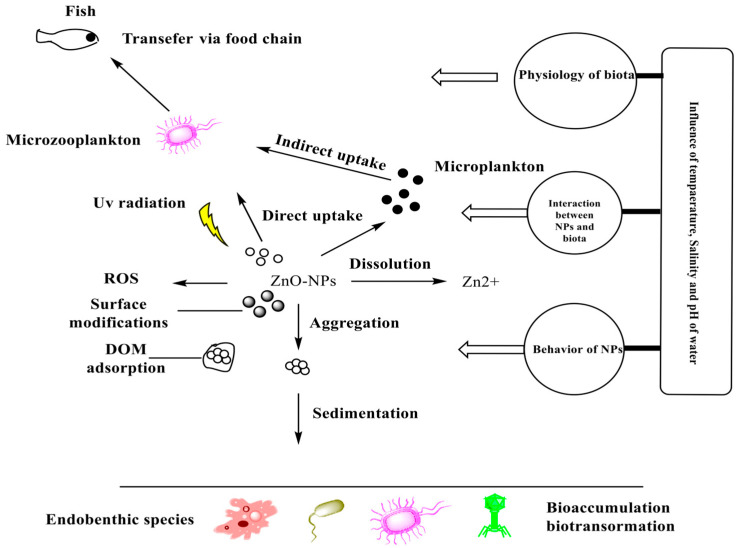
Illustration showing the transformation, transport, and ecological interactions of zinc oxide nanoparticles (ZnO NPs) in the marine environment, including their aggregation, dissolution, sedimentation, and potential impacts on aquatic organisms and ecosystems. Permissions ref [252].

To mitigate the risks associated with ZnO NPs, regulatory bodies have implemented guidelines and exposure limits. For example, the Occupational Safety and Health Administration (OSHA) has established permissible exposure limits (PELs) for ZnO dust and fumes in workplace environments. Similarly, the European Chemicals Agency (ECHA) has issued guidelines on the safe handling and use of ZnO NPs, stressing the importance of appropriate protective equipment and exposure monitoring to safeguard workers’ health [255]. To reduce the toxicity of ZnO NPs, researchers are exploring green synthesis methods that utilize environmentally friendly materials and processes. Additionally, surface modifications of ZnO NPs, such as coating them with biocompatible polymers, can reduce their reactivity and potential toxicity. One study highlighted the success of surface-modified ZnO NPs in reducing cytotoxicity while maintaining their antimicrobial properties [256]. The concept of ‘safe-by-design’ involves designing ZnO NPs with safety considerations integrated from the outset. This approach includes selecting safer raw materials, optimizing particle size, and minimizing the release of toxic ions. Safe-by-Design strategies are being advocated to ensure that ZnO NPs can be safely employed in various applications without posing significant risks to health and the environment [257]. Figure 10 provides insight into the complex fate and behavior of zinc oxide nanoparticles (ZnO NPs) in marine environments, emphasizing their physicochemical transformations and ecological interactions. Once released into seawater, ZnO NPs undergo various processes such as aggregation, sedimentation, and partial dissolution into Zn^2+^ ions, influenced by environmental parameters like pH, salinity, and organic matter content. These transformations affect their mobility, bioavailability, and toxicity. Dissolved zinc ions can disrupt the cellular processes in marine organisms, while particulate forms may interact with plankton, invertebrates, and benthic species, potentially leading to bioaccumulation and trophic transfer. Moreover, ZnO NPs can induce oxidative stress and membrane damage in aquatic organisms, posing ecological risks. Understanding their environmental behavior is crucial for assessing potential long-term impacts on marine biodiversity and for developing strategies to regulate nanoparticle pollution in aquatic ecosystems.

Table 9 presents a comprehensive overview of the toxicity and safety concerns associated with zinc oxide nanoparticles (ZnO NPs), emphasizing their dose-dependent cytotoxicity and potential risks to both human health and the environment. Studies indicate that at higher concentrations or with prolonged exposure, ZnO NPs can induce oxidative stress, DNA damage, mitochondrial dysfunction, and apoptosis in various mammalian cell lines. Additionally, their ability to dissolve into Zn^2+^ ions contributes to toxicity by disrupting cellular ion homeostasis and interfering with enzyme functions. In vivo studies in animal models have also shown accumulation in vital organs, raising concerns about chronic exposure and systemic toxicity. Furthermore, their ecotoxicological impact on aquatic life and soil microorganisms underscores the need for careful evaluations of their environmental release. These findings highlight the importance of establishing standardized safety guidelines, optimizing surface modifications to reduce toxicity, and conducting long-term biocompatibility assessments before the widespread application of ZnO NPs in the biomedical, industrial, and environmental fields.

## 4. Challenges and Future Perspectives

The diverse and evolving applications of zinc oxide nanoparticles (ZnO NPs) make them a cornerstone of nanotechnology research with immense potential for future advancements. Building on the innovations and findings highlighted in this review, future research should focus on addressing existing challenges, enhancing functionality, and exploring new domains of application. While significant progress has been made in the surface modification and doping of ZnO NPs, further exploration of advanced functionalization methods is essential. Future work should aim at developing multi-functional nanoparticles that combine improved stability, biocompatibility, and targeted delivery capabilities. Strategies like biomimetic coatings and responsive surfaces could open up new avenues in medicine and environmental remediation. ZnO NPs have already demonstrated great potential in targeted drug delivery, antimicrobial coatings, and gene therapy. However, translating these findings into clinical applications requires more extensive preclinical and clinical trials. Research should prioritize designing ZnO-based nanomedicines that minimize toxicity while maximizing therapeutic efficacy. Furthermore, integrating ZnO NPs with other nanomaterials could lead to hybrid systems with a superior performance in diagnostics and treatment. With their promising role in water purification and air quality improvement, ZnO NPs are well-positioned to contribute to sustainable environmental solutions. Future research should focus on developing cost-effective and scalable methods for integrating ZnO NPs into purification systems. In the energy sector, optimizing ZnO-based materials for dye-sensitized solar cells and supercapacitors can significantly enhance energy conversion and storage efficiencies, aiding the transition to renewable energy. The growing use of ZnO NPs raises concerns about their potential cytotoxicity and environmental impact. To ensure their safe use, future studies should focus on “safe-by-design” approaches, emphasizing eco-friendly synthesis and surface modifications that reduce toxicity. Long-term studies on the bioaccumulation and environmental persistence of ZnO NPs are also necessary to establish comprehensive safety guidelines. The agricultural sector can benefit substantially from ZnO-NP-based technologies, particularly in the development of nano-fertilizers and pest control agents. Future research should aim to optimize these applications to enhance crop yields while minimizing environmental risks. Precision agriculture technologies incorporating ZnO NPs could also revolutionize farming practices by enabling real-time monitoring and controlled nutrient delivery. The integration of ZnO NPs with emerging technologies such as artificial intelligence, Internet of Things (IoT), and robotics could unlock new possibilities. For instance, smart sensors embedded with ZnO NPs could enhance real-time environmental monitoring, while AI-driven modeling could accelerate the design of nanoparticles with tailored properties. To translate research into real-world applications, clear regulatory frameworks and commercialization pathways are essential. Collaboration between researchers, policymakers, and industry stakeholders will be crucial for ensuring the safe and effective deployment of ZnO-NP-based technologies. The future of ZnO NPs is bright and multifaceted, with the potential to address critical global challenges in healthcare, energy, environment, and agriculture. By leveraging interdisciplinary approaches and prioritizing sustainability, ZnO NPs can continue to advance as a transformative force in science and technology.

### Limitations in Clinical and Field Applications

Despite the broad applicability of zinc oxide nanoparticles (ZnO NPs) across the biomedical, environmental, and agricultural domains, several limitations hinder their translation from laboratory research to clinical and field applications. Toxicological concerns remain a primary barrier, as the long-term effects of ZnO NP accumulation in human tissues and the environment are not fully understood. Cytotoxicity, genotoxicity, and oxidative stress induced by ZnO NPs vary significantly depending on size, shape, surface charge, and dosage, necessitating rigorous biosafety assessments. In clinical settings, challenges such as non-specific biodistribution, immune clearance, and poor targeting efficiency reduce the therapeutic efficacy of ZnO-NP-based drug delivery and cancer therapies. Moreover, regulatory hurdles and a lack of standardized protocols for nanoparticle characterization and safety testing impede clinical approval. In agriculture and environmental fields, inconsistent performances under variable real-world conditions (e.g., soil pH, microbial interactions, and light exposure) can diminish the anticipated benefits of ZnO NPs in pest control, nutrient delivery, and pollutant remediation. Scale-up limitations also pose challenges. The reproducible, cost-effective, and eco-friendly mass production of uniformly dispersed ZnO NPs with desirable surface functionalities remains technically demanding. Furthermore, regulatory frameworks and public perception play a critical role. The absence of well-defined global guidelines for nanoparticle usage, labeling, and disposal raises concerns about environmental release, bioaccumulation, and food chain entry. Addressing these limitations requires a multidisciplinary approach involving materials science, toxicology, environmental engineering, and policymaking to ensure that ZnO-NP-based technologies are both effective and safe for widespread use.

## 5. Conclusions

Zinc oxide nanoparticles (ZnO NPs) have emerged as versatile and multifunctional materials with wide-ranging applications across several fields, including biomedicine, environmental remediation, agriculture, and energy. Their unique properties, such as high surface area, photocatalytic activity, antimicrobial effects, and biocompatibility, make them invaluable tools for addressing complex global challenges. Recent advancements in ZnO NP research, including surface modifications, doping, and the development of nanocomposites, have significantly enhanced their functionality, leading to promising innovations in targeted drug delivery systems, antimicrobial coatings, and sustainable energy solutions. In the biomedical field, ZnO NPs have shown great potential in drug delivery, tissue engineering, and vaccine development, offering targeted therapies and improved therapeutic efficacy. In environmental applications, ZnO NPs have proven effective in water purification, air quality improvement, and pollution control, contributing to sustainable development efforts. Additionally, in agriculture, ZnO NPs enhance plant growth, protect against diseases and pests, and improve crop yields, making them an important tool for sustainable farming practices. Despite their promising applications, concerns regarding the toxicity and environmental impact of ZnO NPs remain. Further research is essential to explore safe, biocompatible formulations and assess the long-term effects of these nanoparticles. Developing robust regulatory frameworks and incorporating safe-by-design strategies will be crucial in ensuring the responsible use of ZnO NPs.

The following points summarize the key insights and future directions for ZnO NPs:

➢ Multifunctionality and Versatility: ZnO NPs offer diverse solutions in various sectors, from healthcare to agriculture and environmental protection, with applications ranging from antimicrobial treatments to pollution control.
➢ Advancements in Biomedical Applications: ZnO NPs are making significant strides in drug delivery, tissue engineering, and vaccine development, offering enhanced therapeutic outcomes.
➢ Environmental and Agricultural Potential: ZnO NPs play a crucial role in environmental remediation and contribute to agricultural productivity, enhancing plant growth and protecting crops.
➢ Challenges in Toxicity and Environmental Impact: While ZnO NPs hold promise, their potential toxicity and long-term environmental impacts warrant further investigation, particularly focusing on safe, biocompatible formulations.
➢ Future Directions and Research: The future of ZnO NPs lies in developing biosafe alternatives, optimizing synthesis methods for large-scale production, and fostering interdisciplinary research to unlock their full potential.

Ultimately, ZnO NPs hold immense promise across various domains, and recent advancements in research have greatly expanded their scope of application. However, to fully realize their potential, ongoing studies must address safety concerns and environmental considerations, ensuring that these nanoparticles are used responsibly and sustainably.

## Figures and Tables

**Table 1:** Recent multifunctional applications of zinc oxide nanoparticles in antimicrobial activities.

Applications	Details	References
Antibacterial		
Medical bandages	ZnO NPs incorporated into bandages to prevent infections in wounds	[35]
Food packaging	Used as coatings to inhibit bacterial growth on perishable food products	[36]
Water filtration systems	Integrated into filters to eliminate waterborne bacterial pathogens	[37]
Antibacterial sprays	Development of surface sprays to reduce microbial contamination	[38]
Coatings for medical devices	Prevent bacterial biofilm formation on catheters and implants	[39]
Antibacterial textiles	ZnO-NP-infused fabrics for odor and infection control	[40]
Oral care products	Added to toothpaste to combat dental plaque and bacterial infections	[41]
Agricultural applications	Prevent bacterial infections in seeds and crops	[42]
Cosmetics	Used in creams for antibacterial properties	[43]
Hand sanitizers	Enhanced with ZnO NPs for extended antimicrobial activity	[44]
Antifungal		
Antifungal coatings	Prevent fungal growth on construction materials	[45]
Seed treatment	Protect seeds from fungal infections	[46]
Food storage	Inhibit fungal spoilage of stored grains and fruits	[47]
Skin infection treatment	Topical formulations for treating fungal skin infections	[48]
Antifungal paints	Applied on walls to prevent mold formation	[49]
Textile industry	Prevent fungal damage to natural fibers	[50]
Crop protection	Prevent fungal diseases in plants like powdery mildew	[51]
Fungal keratitis treatment	Used in eye drops to treat fungal keratitis	[52]
Medical equipment	Used in coatings to reduce fungal contamination	[53]
Pharmaceutical applications	Formulated into antifungal drugs	[54]
Antiviral		
COVID-19 surface coatings	Reduce viral spread on frequently touched surfaces	[55]
Hand sanitizers	Enhanced with ZnO NPs for antiviral efficacy	[25]
Antiviral fabrics	Incorporated into masks to block viral particles	[56]
Treatment of herpes infections	Topical formulations to manage Herpes Simplex Virus	[57]
Influenza virus inhibition	ZnO NPs studied for their effect on reducing viral replication	[58]
Antiviral creams	Effective in managing skin-related viral infections	[59]
Dental antiviral applications	Added to dental materials to prevent viral infections in oral cavities	[60]
Antiviral sprays	Development of surface disinfectants for public spaces	[61]
Preventing Hepatitis B infection	Investigated for reducing HBV replication	[62]
Zika virus inhibition	Studied for their effect on mosquito-borne viral diseases	[63]

**Table 2:** Recent multifunctional applications of zinc oxide nanoparticles in drug delivery.

Application Area	Specific Application	Details	Reference
Targeted Drug Delivery	Cancer Therapy	ZnO NPs conjugated with peptides for targeted drug delivery in prostate cancer cells	[76]
	Antibiotic Delivery	ZnO NPs functionalized with ampicillin show enhanced antibacterial activity	[77]
	Gene Delivery	ZnO NPs for delivering plasmid DNA into mammalian cells for genetic disorder treatment	[78]
Controlled Release and Drug Loading	pH-Responsive Drug Release	ZnO NPs loaded with doxorubicin show controlled release at acidic pH in tumor environments	[79]
	High Drug Loading Capacity	Modified ZnO NPs used for high loading and controlled release of hydrophobic drugs	[80]
	Multifunctional Drug Delivery Systems	ZnO NPs combined with fluorescent dyes for dual drug delivery and bioimaging	[81]
Overcoming Drug Resistance	Reversing Multidrug Resistance in Cancer	ZnO NPs block efflux pumps to enhance drug accumulation in drug-resistant breast cancer cells	[82]
	Enhancing Antibiotic Efficacy	ZnO NPs disrupt bacterial membranes to potentiate the effect of tetracycline against MRSA	[83]
	Combination Therapy	ZnO NPs co-loaded with paclitaxel and resveratrol for treating multidrug-resistant cancers	[84]

**Table 3:** Recent multifunctional applications of zinc oxide nanoparticles in cancer treatment.

Application Area	Specific Application	Details	Reference
Photodynamic Therapy (PDT)	Mechanism of Action	ZnO NPs generate ROS under UV light, inducing apoptosis in cancer cells	[92]
	Improved Targeting	Functionalized ZnO NPs improve localization in tumor tissues for PDT	[93]
	Enhanced Photosensitivity	ZnO NPs combined with natural photosensitizers improve ROS generation	[94]
Chemotherapy Enhancement	Combination with Cisplatin	ZnO NPs reduce cisplatin resistance in ovarian cancer cells	[95]
	Enhanced Doxorubicin Delivery	ZnO NPs increase doxorubicin uptake in multidrug-resistant cancer cells	[96]
	Reduced Drug Toxicity	ZnO NPs lower the systemic toxicity of chemotherapy drugs	[78]
	Co-delivery of Multiple Drugs	ZnO NPs deliver doxorubicin and paclitaxel simultaneously for synergistic effects	[96]
Nanotheranostics	Real-Time Tumor Imaging	ZnO NPs functionalized with fluorescent dyes enable imaging-guided therapy	[97]
	Dual-Mode Imaging	ZnO NPs integrated with MRI contrast agents for precise tumor localization	[78]
	Drug-Activated Imaging	ZnO NPs release imaging agents upon drug delivery for real-time monitoring	[98]
Targeting Specific Cancer Types	Breast Cancer Therapy	ZnO NPs functionalized with antibodies target HER2-positive breast cancer cells	[99]
	Lung Cancer Treatment	ZnO NPs loaded with gefitinib improve targeting of lung adenocarcinoma	[100]
	Glioblastoma Treatment	ZnO NPs penetrate the blood–brain barrier to treat glioblastoma	[101]
Reduction of Side Effects	Minimizing Chemotherapy-Induced Nausea	ZnO NPs decrease the systemic release of chemotherapy agents causing adverse effects	[78]
	Better Biodegradability	ZnO NPs exhibit excellent biodegradability, reducing long-term toxicity	[102]

**Table 4:** Recent multifunctional applications of zinc oxide nanoparticles in vaccine development.

Application Area	Specific Application	Details	Reference
Adjuvant Activity	Mechanism of Adjuvant Action	ZnO NPs stimulate cytokine production, enhancing vaccine potency	[113]
	Improving Vaccine Efficacy	ZnO NPs as adjuvants showed superior results in influenza vaccine trials	[114]
	Safety and Biocompatibility	ZnO NPs exhibit biocompatibility, reducing adverse effects in preclinical vaccine studies	[115]
Antigen Delivery Systems	Enhanced Stability of Antigens	ZnO NPs protect protein antigens from enzymatic degradation, increasing their shelf life	[116]
	Targeted Delivery	ZnO NPs functionalized with peptides target lymphatic tissues effectively	[117]
	Controlled Release of Antigens	ZnO NPs enable the gradual release of antigens, leading to sustained immune activation	[115]
New Vaccine Development	COVID-19 Vaccines	ZnO NPs aid in stable delivery of mRNA-based COVID-19 vaccines	[118]
	Vaccines for Emerging Infectious Diseases	ZnO NPs are being evaluated for Zika and SARS-CoV-2 combination vaccines	[119]
	Multivalent Vaccines	ZnO NPs contribute to simultaneous antigen delivery for influenza and RSV vaccines	[120]

**Table 5:** Recent multifunctional applications of zinc oxide nanoparticles in environmental remediation.

Application Area	Specific Application	Mechanism/Functionality	Key Findings/Outcomes	References
Water Purification	Degradation of Organic Pollutants	Photocatalysis under UV light	Efficient removal of textile dyes and phenolic compounds from wastewater	[129]
	Adsorption of Heavy Metals	High affinity for metal ions on nanoparticle surfaces	Removal of cadmium (Cd) and chromium (Cr) from industrial wastewater	[130]
	Disinfection of Pathogens	Reactive oxygen species generation causing microbial inactivation	Successful elimination of *Escherichia coli* and *Cryptosporidium* from water systems	[131]
Air Purification	Photocatalytic Degradation of VOCs	Oxidation of harmful organic compounds into non-toxic byproducts	Reduction in indoor air pollutants, including formaldehyde and benzene	[132]
	Removal of Particulate Matter	Aggregation of particles facilitated by surface interactions	Enhanced capture of particulate matter in urban environments using ZnO coatings on filters	[133]
	Inactivation of Airborne Pathogens	Antimicrobial activity through surface interactions and ion release	Suppression of bacterial growth in hospital and industrial ventilation systems	[134]
Soil Remediation	Immobilization of Heavy Metals	Chemical binding and stabilization of toxic metals in soil	Immobilization of lead (Pb) and arsenic (As), reducing leaching risks	[135]
	Degradation of Organic Pollutants in Soils	UV-activated degradation of pesticides and herbicides	Reduction in chlorpyrifos and atrazine residues in agricultural soils	[135]
	Enhancement of Soil Quality	Stimulation of microbial activity and nutrient cycling	Improved soil fertility and crop productivity in treated agricultural fields	[136]

**Table 6:** Comparative performance of ZnO-based gas sensors.

Sensing Material	Target Gas	Operating Temp (°C)	Response/Recovery Time (s)	Limit of Detection (LOD)	Reference
Pure ZnO Nanorods	NO_2_	Room temperature	60/80	1 ppm	[146]
ZnO Nanowires	NO_2_	Room temp	40/55	0.5 ppm	[147]
ZnO–SnO_2_ Heterostructure	Ethanol	250	12/18	10 ppm	[152]
MoS_2_/ZnO Heterostructure	NO_2_	Room temp	10/13	100 ppb	[153]
MoS_2_/ZnO Heterostructure	NO_2_ (UV-aided)	Room temp	7/11	50 ppb	[154]
MoS_2_/ZnO Hierarchical	H_2_S	Room temp	9/14	0.5 ppm	[155]

**Table 7:** Recent multifunctional applications of zinc oxide nanoparticles in electronics, optoelectronics, and different industrial sectors.

Application	Description	Recent Studies
Electronics and Optoelectronics		
Transparent Conductive Films	ZnO NPs used in displays, touch screens, and solar cells as cost-effective alternatives to ITO	[184]
Gas Sensors	High-sensitivity gas sensors for environmental monitoring and industrial safety	[185]
Light-Emitting Diodes (LEDs)	High-efficiency LEDs in UV and visible light spectra	[186]
Photodetectors	UV light detection for environmental monitoring and medical diagnostics	[78]
Piezoelectric Devices	Energy-harvesting devices for wearable electronics	[187]
Flexible Electronics	ZnO NPs in wearable sensors and foldable displays for enhanced flexibility	[188]
Transistors	Thin-film transistors for next-generation electronic devices	[189]
Memory Devices	Non-volatile memory devices like RRAM with low power consumption	[190]
Solar Cells	Electron transport layers in perovskite solar cells for improved efficiency	[191]
Thin-Film Coatings	Protective coatings for electronic devices to enhance UV protection and durability	[192]
Textiles		
Antimicrobial Textiles	Fabrics with long-lasting antimicrobial protection for medical and hygiene products	[193]
UV-Blocking Fabrics	Textiles that block harmful UV rays, ideal for outdoor clothing	[194]
Self-Cleaning Textiles	Fabrics with photocatalytic self-cleaning properties	[195]
Moisture-Wicking Fabrics	Enhanced breathability for activewear and outdoor gear	[196]
Flame-Retardant Fabrics	Textiles with improved safety through flame-retardant properties	[197]
Anti-Odor Fabrics	Fabrics that prevent odor caused by bacteria, popular in socks and underwear	[40]
Enhanced Dyeing	Improved dyeability and color fastness for vibrant and durable colors	[198]
Thermal Regulation	Textiles that regulate temperature, useful in sportswear and military uniforms	[199]
Anti-Static Fabrics	Fabrics with reduced static electricity, beneficial in electronic work environments	[200]
Durability Enhancement	Increased resistance to wear and tear, extending garment lifespan	[201]
Improved bonding	Enhanced nanoparticle adhesion and durable antimicrobial finishing	[202]
Cosmetics		
Sunscreens	Broad-spectrum UV protection in daily-use sunscreens	[203]
Anti-Aging Creams	Prevention of photoaging and promotion of collagen synthesis	[204]
Acne Treatment	Reduction in acne-causing bacteria and soothing of inflamed skin	[205]
Moisturizers	Enhanced skin hydration and barrier function, ideal for dry and sensitive skin types	[206]
Makeup Products	Mattifying and skin-tone-evening effects in foundations and powders	[172]
Deodorants	Long-lasting freshness without harsh chemicals	[207]
Toothpaste	Prevention of cavities, gum disease, and bad breath in dental care products	[170]
Hair Care Products	Scalp soothing, dandruff reduction, and UV protection in shampoos and conditioners	[208]
Antiperspirants	Reduction in sweat and bacteria responsible for body odor	[209]
Wound Healing Creams	Acceleration of healing and reduction in infection risk in wound care	[210]
Food Packaging		
Antimicrobial Packaging	Prevention of microbial growth to extend the shelf life of food products	[36]
UV-Blocking Packaging	Protection of food products from photodegradation and spoilage	[211]
Oxygen Scavenging	Reduction in oxygen levels to slow down oxidation and spoilage	[212]
Ethylene Removal	Removal of ethylene gas to extend the shelf life of fresh produce	[213]
Active Biodegradable Packaging	Sustainable packaging materials with antimicrobial and UV-blocking functions	[211]
Nanocomposite Films	Enhanced mechanical strength and barrier properties in food packaging materials	[214]
Smart Packaging	Real-time monitoring of food freshness through changes in temperature, pH, or gas composition	[180]
Odor-Absorbing Packaging	Neutralization of unwanted odors released by certain foods during storage	[215]
Improved Barrier Properties	Enhanced protection against moisture, gases, and oils	[216]
Edible Coatings	Edible coatings that maintain freshness and extend the shelf life of fresh fruits and vegetables	[217]
Biofilm Development	Biomedical applications such as topical dressings or as packaging for the food industry.	[218,219]

**Table 8:** Recent multifunctional applications of zinc oxide nanoparticles in agriculture.

Application Area	Description	Example/Study	Reference
Antimicrobial Activity	ZnO NPs protect seeds and crops from pathogens by inhibiting microbial growth	Inhibition of *Pseudomonas syringae* in various crops	[236,237]
Fertilizer Efficiency Enhancement	ZnO NPs improve nutrient uptake and enhance crop growth by acting as a smart fertilizer component	Increased growth and yield in wheat and maize due to improved root development	[236,238]
Pest and Disease Management	ZnO NPs serve as a dual-action agent by protecting crops from pathogens and repelling insects	Formulation of ZnO NPs in sprays for fungal and bacterial infection protection and insect repulsion	[230]
Soil Health Improvement	ZnO NPs provide a slow-release source of zinc, essential for soil fertility and plant health	Improved soil fertility in zinc-deficient soils and better nutrient availability	[239,240]
UV Protection for Crops	ZnO NPs act as UV shields, protecting plants from excessive UV radiation	Application of ZnO NPs to tomato and strawberry plants to reduce UV stress	[241,242]
Seed Germination and Growth Promotion	ZnO NPs enhance seed germination and early plant growth by promoting water uptake and enzyme activity	Faster germination and more robust seedlings in wheat treated with ZnO NPs	[243,244]
Water Management	ZnO NPs are used in materials for soil moisture retention, aiding in efficient water use in agriculture	Development of ZnO-NP-based materials for consistent water supply to crops during dry periods	[245]
Nano-Encapsulation of Agrochemicals	ZnO NPs are used to encapsulate agrochemicals for targeted delivery and reduced environmental impact	Nano-encapsulation of herbicides and insecticides using ZnO NPs for enhanced efficacy	[246]
Biotic and Abiotic Stress Tolerance	ZnO NPs enhance crop tolerance to biotic and abiotic stresses, such as pathogen attack, drought, and salinity	Application of ZnO NPs to soybean for drought resistance and to rice for salinity tolerance	[127,247]
Nanobiosensors for Precision Agriculture	ZnO NPs are incorporated into nanobiosensors for real-time monitoring of soil health, nutrient levels, and plant stress	Development of nanobiosensors using ZnO NPs for optimizing agricultural inputs in precision farming	[248,249]

**Table 9:** Toxicity and safety concerns of ZnO NPs.

Area	Concerns	Examples/Studies	References
Human Health Risks	Cytotoxicity, genotoxicity, respiratory toxicity, dermal toxicity	DNA damage in human lung cells; respiratory diseases in workers; skin penetration in damaged skin	[258]
Environmental Impact	Ecotoxicity in aquatic systems, soil toxicity, bioaccumulation	Inhibition of algal growth; reduction in soil microbial diversity; bioaccumulation in aquatic organisms	[259,260]
Safety Measures and Regulations	Guidelines, green synthesis, surface modifications, safe-by-design approaches	OSHA exposure limits; surface-modified ZnO NPs; Safe-by-Design strategies	[261,262]
